# Larval crowding accelerates *C*. *elegans* development and reduces lifespan

**DOI:** 10.1371/journal.pgen.1006717

**Published:** 2017-04-10

**Authors:** Andreas H. Ludewig, Clotilde Gimond, Joshua C. Judkins, Staci Thornton, Dania C. Pulido, Robert J. Micikas, Frank Döring, Adam Antebi, Christian Braendle, Frank C. Schroeder

**Affiliations:** 1 Molecular Prevention Group, Institute of Human Nutrition and Food Science, Christian-Albrechts-University, Kiel, Germany; 2 Boyce Thompson Institute and Department of Chemistry and Chemical Biology, Cornell University, Ithaca, NY, United States of Ameirca; 3 Institut de Biologie Valrose, CNRS, UMR7277, Parc Valrose, Nice, France; 4 Department of Molecular Genetics of Ageing, Max Planck Institute for Biology of Ageing, and Cologne Excellence Cluster on Cellular Stress Responses in Aging-Associated Diseases (CECAD), University of Cologne, Cologne, Germany; Stanford University, UNITED STATES

## Abstract

Environmental conditions experienced during animal development are thought to have sustained impact on maturation and adult lifespan. Here we show that in the model organism *C*. *elegans* developmental rate and adult lifespan depend on larval population density, and that this effect is mediated by excreted small molecules. By using the time point of first egg laying as a marker for full maturity, we found that wildtype hermaphrodites raised under high density conditions developed significantly faster than animals raised in isolation. Population density-dependent acceleration of development (Pdda) was dramatically enhanced in fatty acid β-oxidation mutants that are defective in the biosynthesis of ascarosides, small-molecule signals that induce developmental diapause. In contrast, Pdda is abolished by synthetic ascarosides and steroidal ligands of the nuclear hormone receptor DAF-12. We show that neither ascarosides nor any known steroid hormones are required for Pdda and that another chemical signal mediates this phenotype, in part via the nuclear hormone receptor NHR-8. Our results demonstrate that *C*. *elegans* development is regulated by a push-pull mechanism, based on two antagonistic chemical signals: chemosensation of ascarosides slows down development, whereas population-density dependent accumulation of a different chemical signal accelerates development. We further show that the effects of high larval population density persist through adulthood, as *C*. *elegans* larvae raised at high densities exhibit significantly reduced adult lifespan and respond differently to exogenous chemical signals compared to larvae raised at low densities, independent of density during adulthood. Our results demonstrate how inter-organismal signaling during development regulates reproductive maturation and longevity.

## Author summary

The nematode *C*. *elegans* is one of the most highly developed models for the elucidation of conserved mechanisms connecting environmental cues to the regulation of animal lifespan and development. Surprisingly, the effects of larval population density on developmental timing and adult lifespan have not been investigated, although population density is known to affect developmental dynamics and survival in many species. We here describe a novel phenotype in *C*. *elegans*: population density-dependent *acceleration* of development. That high population density would accelerate development is unexpected, since at high population density accumulation of dauer pheromone, a developmental arrest signal, would be expected to slow down development. However, we found that *C*. *elegans* development is regulated by a pull-push mechanism, based on at least two different types of pheromone-like signals: the developmental acceleration signal we first describe in this manuscript, and its antagonist, the dauer pheromone, whose chemical make-up has gradually emerged over the past 10 years. We further show that both developmental acceleration and deceleration are mediated by two nuclear hormone receptors that have close mammalian homologs. Finally we demonstrate that larval population density predetermines adult lifespan in *C*. *elegans* hermaphrodites, including responses to hormonal stimuli during adulthood.

## Introduction

Sensing environmental conditions is of central importance for organismal development and survival and has been recognized as a major driver of adaptive evolution [1,2]. The nematode *Caenorhabditis elegans* is a particularly useful model for studying the effects of complex environmental inputs on development and lifespan [3,4], and *C*. *elegans* has become one of the most important models for studying conserved mechanisms of aging. Several lines of evidence indicate that development and lifespan in *C*. *elegans* are controlled by interorganismal signaling. High population density under conditions of dietary restriction triggers arrest of development as dauer larvae, a long-lived, highly stress resistant alternate larval stage that can persist under adverse environmental conditions for many months [4]. The dauer-inducing population density signal was shown to consist of a synergistic mixture of small molecules, the ascarosides [5–7]. Perception of dauer-inducing ascarosides downregulates insulin, cGMP and TGF-β signaling, which in turn downregulates expression of enzymes involved in the biosynthesis of steroid hormones called dafachronic acids (DAs), the endogenous ligands of the nuclear receptor DAF12, a homolog of vertebrate vitamin D and liver-X receptors [8,9].

Binding of DAs to DAF-12 is required for normal reproductive development, whereas abolishment of DA biosynthesis results in unliganded DAF-12 triggering larval arrest at the long-lived and non-feeding dauer stage [9,10]. Parallel work showed that *C*. *elegans* and other nematodes produce a large diversity of ascaroside-based small molecules, the nematode-derived modular metabolites (NDMMs), which are derived from combinatorial assembly of chemical building blocks from lipid, amino acid, carbohydrate, citrate, neurotransmitter, and nucleoside metabolism [7,11]. NDMMs control many aspects of nematode life history, including mating and aggregation behaviors as well as adult lifespan.

Whereas dauer formation and associated signaling pathways have been investigated in great detail, other effects of population density on developmental progression and adult lifespan remain poorly understood. For example, the lifespan of *C*. *elegans* males starkly decreases at high worm densities, and the presence of male worms has been shown to accelerate aging in hermaphrodite worms [12,13]. Furthermore, decreased sensory stimulation at low worm densities has been shown to slow down development and the begin of egg laying [14]. Based on serendipitous observations during worm synchronization for aging studies, we hypothesized that population density may regulate the rate of developmental maturation in *C*. *elegans* via an excreted chemical signal. Using the time point of first egg laying as a marker for full developmental maturity we show that developmental rate in *C*. *elegans* is controlled by a push-pull mechanism that relies on several different types of interorganismal small-molecule signals that are excreted by the worms. We show that high population conditions accelerate development by an as-yet unidentified chemical signal, and that this effect is counteracted by specific ascaroside pheromones and DAs. In addition, we found that population density experienced during larval development strongly affects adult lifespan.

## Results

### Population density affects rate of developmental progression

**Fig 1:**
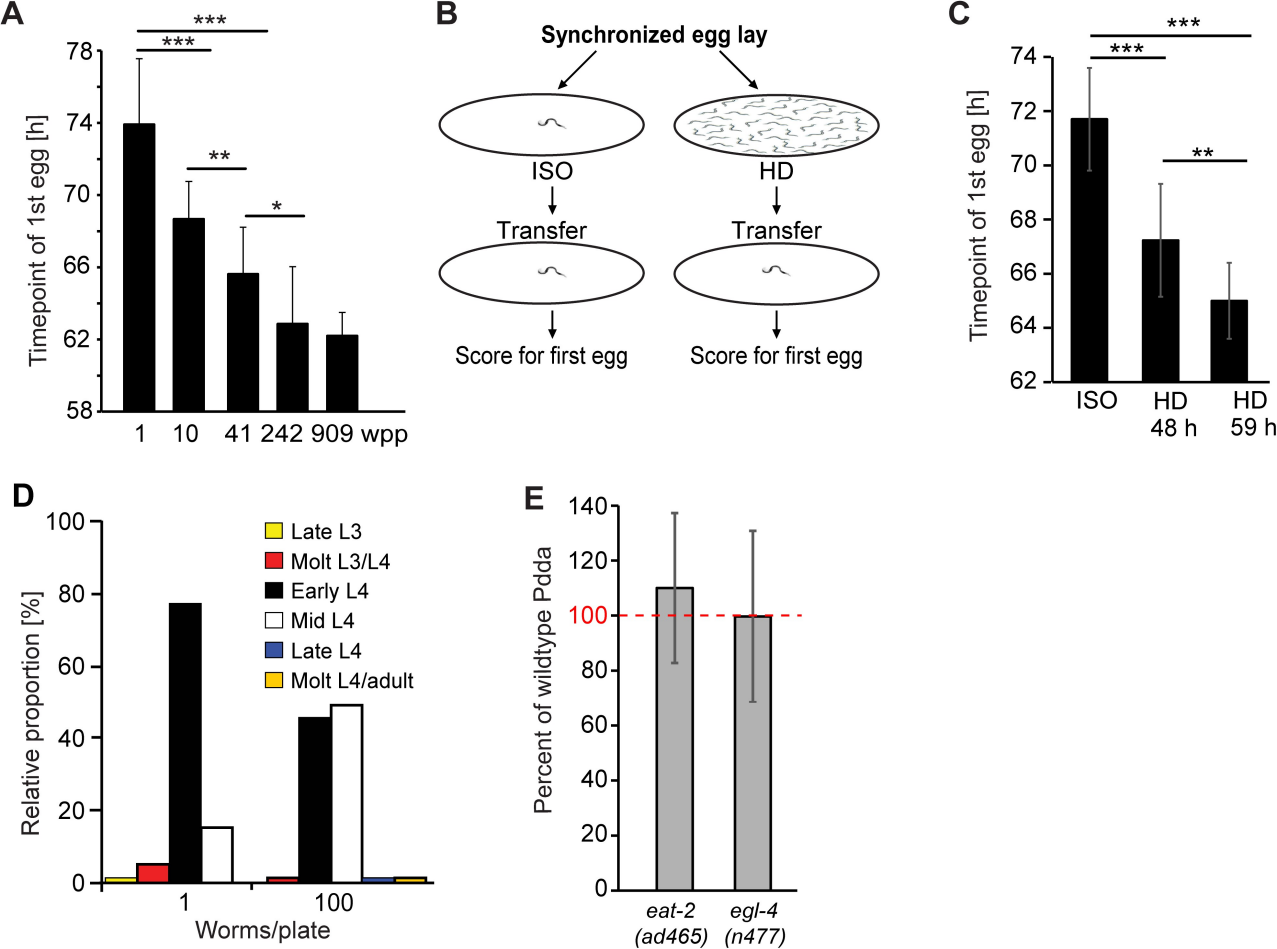
Population density dependent acceleration of development (Pdda) in *C*. *elegans* hermaphrodites. **A)** Time point of first egg laying on plates with different population densities, set up according to protocol A (see Methods). First egg laying was scored from 60 h after plating. **B)** Schematic of experimental protocol B used for measuring the effect of population density on the time point of first egg laying. **C)** Time point of first egg laying of worms isolated throughout development (ISO) or kept at high density for 48 h or 59 h (HD) before transfer onto new plates at one worm per plate. **D)** Developmental stage of isolated worms and groups of 100 worms at 52 h. **E)** Pdda is unaffected by mutation of *eat-2 and egl-4*, (see Methods for calculating percent wildtype Pdda). Error bars, STD; ***P < 0.0001; **P < 0.001; *P < 0.05.

To facilitate comparing developmental rates across different conditions and genotypes, we considered using the time point of first egg laying as an easily observable marker for reproductive maturation. Egg laying has been used previously as part of a suite of assays to characterize the effects of decreased sensory stimulation on behavior and neuronal development in *C*. *elegans* [14]. In an initial experiment, we measured the time point of first egg laying on plates with 1 to ~900 worms per plate (Fig 1A, S1 Table). Results from this assay suggested that the time point of first egg lay is strongly dependent on the number of worms per plate; however, the chance of observing an egg on a plate obviously increases with the number of worms per plate, which made it difficult to ascertain the significance of the effect.

Therefore, we established a more elaborate protocol (Fig 1B) in which we raised worms from eggs, either in isolation (one worm per plate, "ISO worms") or on high density plates ("HD worms", raised at ~100 worms/plate), and then transferred both ISO and HD worms at late L4 larval stage (after 59 h development on ISO or HD plates), a few hours before egg laying would start, onto fresh plates, at one worm per plate. Using this protocol we confirmed that HD worms started egg laying significantly earlier than ISO worms (Fig 1C, S1 Table), whereby the magnitude of the effect depends on the amount of time the worms were kept at different density before transfer (Fig 1C, S1 Fig, S1 Table, S2 Table). To assess whether population density-dependent egg laying is associated with overall faster development of HD worms, we compared vulva development and outgrowth of the gonad arms of ISO and HD worms 52 h after hatching. Whereas ISO worms were predominantly in the early larval stage 4 (L4), most HD worms had developed to mid L4 stage (Fig 1D, S1 Fig, S3 Table), corresponding to a 2 to 3 h developmental lead of HD animals. We also tested whether ISO and HD worm differed with regard to embryo volume and overall fecundity. We found that the total number of progeny on the first day of egg laying was increased in HD worms (S2 Fig); however, total numbers of eggs laid were similar for ISO and HD worms (S2 Fig). Taken together, these observations suggest that earlier egg laying of worms raised at high densities is indicative of overall faster development. We refer to this phenotype as **p**opulation **d**ensity-**d**ependent **a**cceleration of development (Pdda).

**Fig 2:**
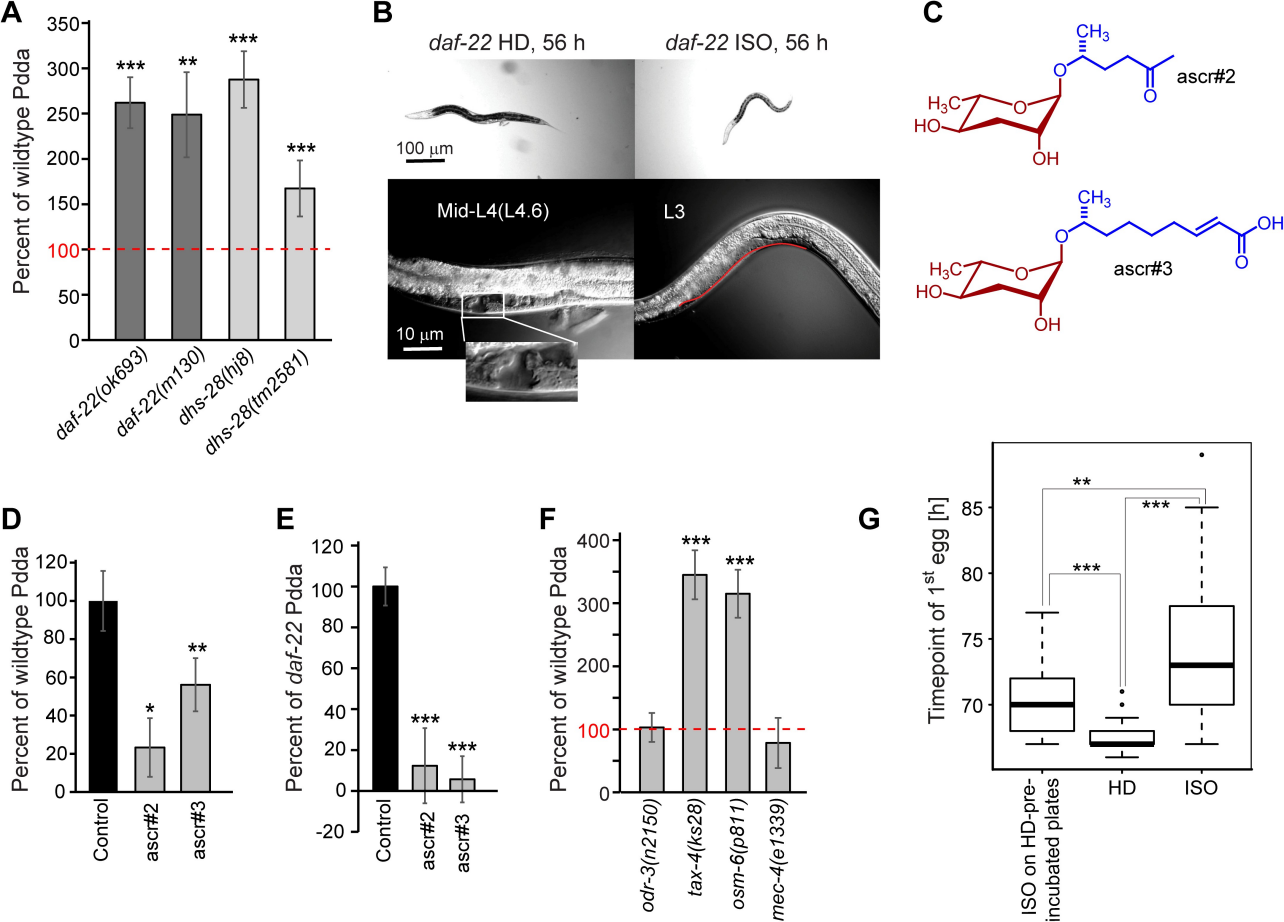
Pdda is counteracted by ascaroside signaling. **A)** Pdda is increased in peroxisomal β-oxidation mutants compared to wildtype. **B)** DIC images of developing *daf-22(ok693)* mutant worms at 56 h, grown under HD (left panels) and ISO (right panels) conditions, showing complete animals (upper panels) and the gonadal tract (lower panels); lower left panel: gonadal arms and vulva (L4.6 stage) in an HD animal (enlargement: magnified vulva); lower right panel: L3 stage gonad, left arm (red highlight) in an ISO animal; **C)** Chemical structures of major ascarosides produced by wildtype hermaphrodites. **D, E)** Ascarosides reduce Pdda in wildtype (**D**) and *daf-22* mutant (**E**) worms. Synthetic ascarosides were added at 400 nM. **F)** Pdda in chemosensory and mechanosensory mutants; **G)** Time point of first egg laying of *daf-22(ok693)* mutants, comparing HD worms, ISO worms, and ISO worms on HD pre-incubated plates. Box plot showing medians, upper and lower quartiles, whiskers at 1.5 IQR. Error bars, STD (panels A, D, E, F); ***P < 0.0001; **P < 0.001; *P < 0.05.

**Fig 3:**
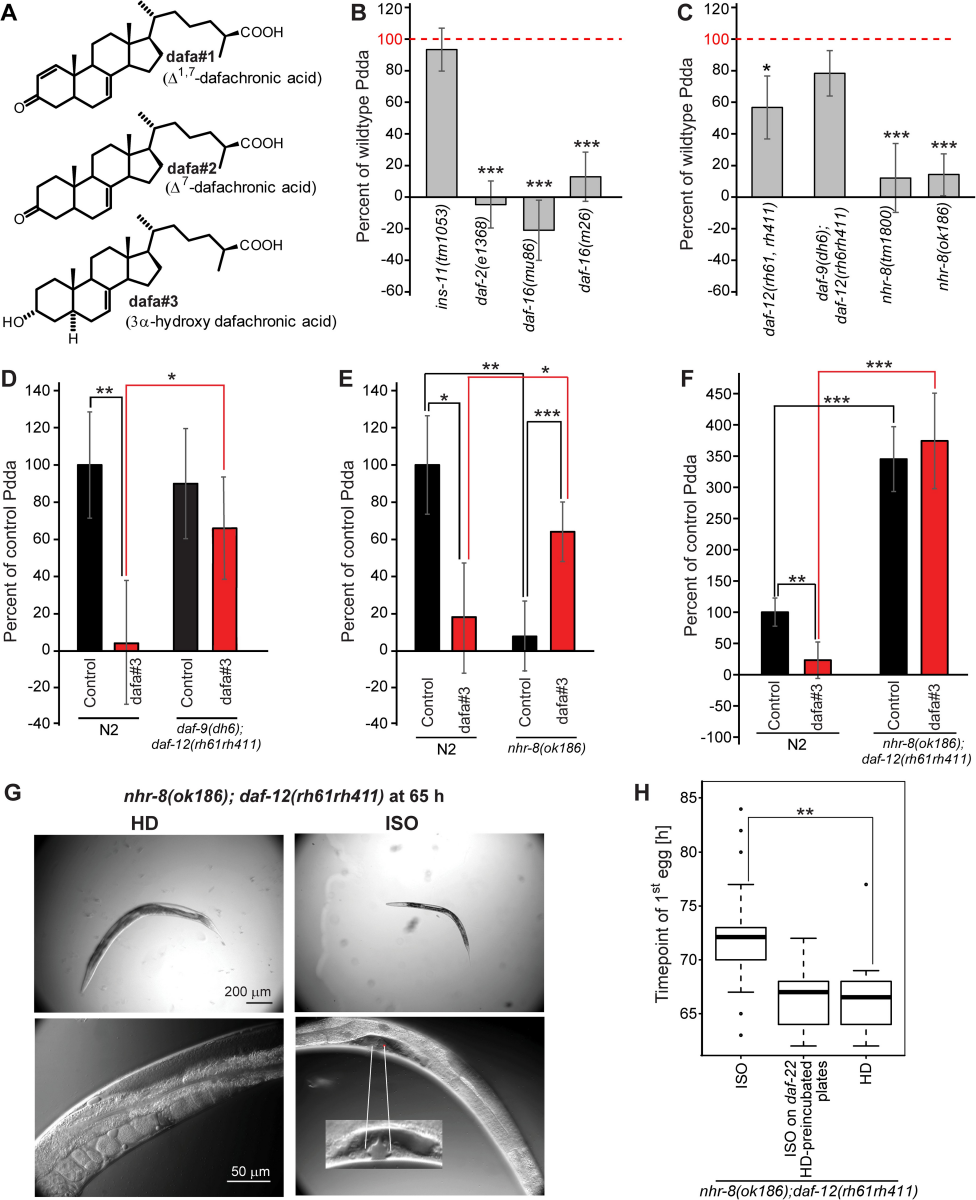
Pdda interacts with steroid hormone and nuclear hormone receptor signaling. **A)** Chemical structures of endogenous DAs. **B)** Pdda in insulin signaling mutants. **C)** Pdda in nuclear hormone receptor signaling mutants. **D)** Effect of dafa#3 (at 100 nM) on Pdda in wildtype and *daf-9; daf-12* double mutants. **E)** Effect of dafa#3 (at 100 nM) on Pdda *nhr-8* mutant worms compared to wildtype. **F)** Effect of dafa#3 (at 100 nM) on Pdda in *nhr-8;daf-12* double mutant worms compared to wildtype. **G)** DIC images of developing *nhr-8;daf-12* double mutant worms at 65 h grown under HD (left panels) and ISO (right panels) conditions, showing complete animals (upper panels) and the gonadal tract (lower panels); lower left panel: right gonadal arm and proliferating oocytes (young adult stage) in an HD animal, right lower panel: L4.6 stage gonad, right gonad arm and vulva in an animal raised under ISO conditions (enlargement: magnified vulva). **H)** Time point of first egg laying of *nhr-8;daf-12* double mutants, comparing HD worms, ISO worms, and ISO worms on plates pre-incubated with *daf-22(ok693)* worms. Box plot showing medians, upper and lower quartiles, whiskers at 1.5 IQR. Error bars, STD (panels B-E); ***P < 0.0001; **P < 0.001; *P < 0.05. The Bonferroni correction was used to assess significance for multiple comparisons in panels D-F.

Next, we screened genes in several pathways related to the regulation of development and lifespan for their potential roles in Pdda. Because developmental times of different mutants vary considerably, we focussed on differences in time points of first egg laying between ISO and HD worms relative to the time difference observed in ISO and HD wildtype worms, which we report in percent of the effect observed for wildtype (Figs 1–3, also see Methods). First, we considered the possibility that differences in food intake may underlie Pdda. Although the bacterial lawn on HD plates did not appear to be significantly depleted by the time worms reached adulthood, it seemed possible that Pdda is caused by differences in feeding behavior or food availability, which have been shown to affect many aspects of development and germline proliferation [15]. Therefore we compared developmental rates of HD and ISO *eat-2* mutant worms, which exhibit reduced pharyngeal pumping rates [16]. We found that, although *eat-2* mutant worms start laying eggs later than wild type, the time difference between the start of egg laying of *eat-2* ISO and HD worms, relative to total time to maturity, is similar to that of wildtype (Fig 1E, S4 Table). Pdda was also unchanged in *egl-4* mutant worms, which are egg laying-defective due to egg retention [14] (Fig 1E, S4 Table), supporting that Pdda is unrelated to egg laying defects or inappropriate egg retention. In addition, Pdda was not affected by mutations of the NADH dependent histone deacetylase SIR-2.1, which may play a role for some aspects of dietary restriction-mediated lifespan changes [3] and is required for ascaroside-mediated increased lifespan and stress resistance [17], the nuclear receptor NHR-49, which is required for entering the starvation-induced adult reproductive diapause (ARD, [18]), and the transcription factor heat shock factor-1 (HSF-1), a general regulator of stress-induced gene expression [19] that is also required for aspects of dietary restriction-mediated lifespan extension [20] (S3 Fig, S4 Table). Taken together, these results suggest that differences in food intake are unlikely to be the primary cause of the observed population-density dependent effects on development.

### Ascaroside signaling counteracts Pdda

We were initially surprised by the finding that *C*. *elegans* develop faster under HD conditions than in isolation, given that dauer-inducing ascarosides accumulate at much higher concentrations on HD plates [7], which we hypothesized would slow down, but not accelerate development. Alternatively, given the wide range of functions that chemically different ascarosides serve in *C*. *elegan s*[11], it also seemed possible that development on HD plates is faster because of accumulation of specific ascarosides. To clarify whether ascaroside production plays a role for Pdda, we investigated the effect of population density on development of *daf-22* and *dhs-28* mutant worms. *daf-22* and *dhs-28* encode two enzymes of the peroxisomal β-oxidation pathway that are required for the biosynthesis of all known ascaroside-based signaling molecules in *C*. *elegans* [21], and therefore *daf-22* and *dhs-28* mutant worms are devoid of all known ascaroside-based signalling molecules. We found that Pdda is starkly increased in *daf-22* and *dhs-28* mutants, with *daf-22* and *dhs-28* HD worms developing 8–17 h faster than ISO mutant worms, compared to a time difference of typically 2–5 h for WT (Fig 2A and 2B, S7 Fig, S5 Table). The strong enhancement of Pdda in ascaroside biosynthetic mutants distinguishes this phenotype from the effect of males on hermaphrodite lifespan (male-induced demise, MID) [13], which is partially abolished in *daf-22* mutants.

Next we tested the effect of adding synthetic ascarosides to both HD and ISO plates. We found that Pdda in WT is partially suppressed by physiological concentrations of the strongly dauer inducing ascaroside ascr#2 [7] (Fig 2C and 2D, S7 Table). Supplementation of ascaroside-defective *daf-22* worms with synthetic ascarosides also suppressed the starkly enhanced Pdda phenotype in this mutant (Fig 2E, S7 Table). These results suggested that accumulation of dauer-inducing ascarosides in the case of wildtype worms results in less Pdda compared to the ascaroside-free mutant *daf-22*.

Ascarosides are perceived via several ciliated sensory neurons [6,22,23], and therefore impairment of chemosensation should also remove the Pdda-suppressing effect of ascarosides. We tested mutants of *osm-6*, which is required for proper cilia formation in sensory neurons [24] and *tax-4*, which encodes a subunit of a cyclic nucleotide-gated channel expressed in ciliated sensory neurons where it localizes to the cilia [25]. Previous work has shown that both *osm-6* and *tax-4* mutants are defective in their responses to dauer-inducing ascarosides [25]. We found that Pdda is strongly increased in both *osm-6* and *tax-4* mutants, to a similar extent as in peroxisomal β-oxidation mutants (Fig 2F, S8 Table). We also showed that in *tax-4* and *osm-6* mutants ascr#2 does not affect Pdda, whereas ascr#2 abolishes Pdda in wildtype (S4 Fig, S9 Table). In contrast, in worms mutant for *odr-3*, which encodes a G-protein alpha subunit that is required for general chemotaxis [26], but not for dauer pheromone perception, Pdda was unchanged compared to wildtype (Fig 2F, S8 Table). Taken together, our results indicate that chemosensation of dauer-inducing ascarosides reduces the effect of population density on developmental rate, and that, in wildtype, dauer-inducing ascarosides partially mask the effects of another population-density dependent factor that accelerates development and represents the proximal cause of Pdda.

Accelerated development at high population density could be due to increased mechanosensory stimulation, which has been shown to affect egg laying [14,27], or result from accumulation of excreted or secreted small molecules other than ascarosides. Pdda was not significantly reduced in mechanosensory defective *mec-4* mutant worms (Fig 2F, S8 Table). To test whether excreted small molecules are responsible for Pdda, we conditioned plates with HD *daf-22* mutant worms (grown from larval stage L1 to L4), removed these worms, and then compared development of *daf-22* ISO worms on HD *daf-22*-conditioned plates with *daf-22* ISO and HD worms on regular plates (Fig 2G, S6 Table). We found that *daf-22* ISO worms on HD-conditioned plates developed faster than *daf-22* ISO worms on regular plates, though not as fast as *daf-22* HD worms. Similarly, isolated wildtype worms grown on plates that have been pre- incubated with N2 larvae started egg laying significantly earlier than isolated N2 worms grown on non pre-incubated plates (S5 Fig, S6 Table). Thus, it appears that one or more compounds excreted by worms on HD plates accelerate development.

### Pdda depends on the nuclear hormone receptor NHR-8

Downstream of ascaroside perception, the insulin/IGF signaling pathway plays a central role in regulating *C*. *elegans* development and lifespan, in part by regulating the biosynthesis of the dafachronic acids (DAs), the steroidal ligands of the nuclear receptor DAF-12 [8,9,28,29]. The DAs are produced from dietary cholesterol via a multi-step enzymatic pathway that is being studied extensively [30]. Three different endogenous DA's have been identified, named dafa#1-dafa#3 (Fig 3A), and binding of DAs to DAF-12 suppresses developmental arrest at the dauer stage and promotes reproductive development [31–33]. Notably, higher concentrations of DA have been shown to accelerate development [34]. One of the last steps of the biosynthesis of the DAs requires the cytochrome P450, DAF-9, and, correspondingly, developing *daf-9* null mutant worms arrest as dauer-like larvae, unless supplied with exogenous DA [35]. DAF-9 expression, and thus DA biosynthesis, is downregulated in mutants of the insulin/insulin-like growth factor receptor DAF-2, whereas DAF-9 expression and DA biosynthesis are increased in knockout mutants of *daf-16*, the FOXO transcription factor acting downstream of *daf-2* [8,34]. We found that Pdda is abolished in both *daf-2* and *daf-16* mutants, in contrast to many other aging- or developmental phenotypes [29] (Fig 3B, S10 Table, S11 Table). We also tested a null mutant of the insulin-like peptide *ins-11*, which is partially required for MID [13]. We found that Pdda is unchanged in *ins-11* mutants, providing additional evidence that hermaphrodite population density affects development in a manner that is distinct from the effects of males on lifespan (Fig 3B, S10 Table). Abolishment of Pdda in *daf-2* as well as *daf-16* mutants argued that while insulin/IGF signaling plays a role, it may not act exclusively via regulation of DA production [8,9,36], which is oppositely regulated in *daf-2* and *daf-16* mutants.

To determine whether DAF-12 or DAs are required for Pdda, we tested the null-allele *daf-12(rh61; rh411)* and the double mutant *daf-9(dh6); daf-12(rh61; rh411)*, which does not produce any DAs. We found that Pdda is similar to wildtype in *daf-9(dh6); daf-12(rh61 rh411)* double mutant worms and slightly reduced in *daf-12(rh61 rh411)* worms (Fig 3C, S12 Table). These results indicated that DA signaling via DAF-12 is not required for Pdda. However, we found that exogenous addition of DAs largely abolished Pdda in wild type as well as the enhanced Pdda in *daf-22* mutant worms (Fig 3D–3F, S7 Fig, S14 Table). Exogenous addition of DAs had no significant effect on Pdda in *daf-9(dh6);daf-12(rh61 rh411)* double mutant worms, indicating that abolishment of Pdda in wildtype is in fact dependent on DAF-12 (Fig 3D, S15 Table).

Recent work has shown that steroid hormone signaling in *C*. *elegans* also involves NHR-8, a nuclear hormone receptor that is closely related to DAF-12. Although DAs are not known to act as ligands of NHR-8, DA signaling through NHR-8 is required for aspects of dietary-restriction-mediated longevity [37] and regulates cholesterol and DA homeostasis [38]. We asked whether NHR-8 plays a role for Pdda, or for abolishment of Pdda by DAs. We found that Pdda is largely abolished in the loss-of-function mutants *nhr-8(ok186)* and *nhr-8(tm1800)* (Fig 3C). Next we tested the effect of one specific DA, dafa#3, on *nhr-8* mutants. Surprisingly, whereas dafa#3 abolished Pdda in wildtype, we found that in *nhr-8* null mutants dafa#3 largely restored Pdda (Fig 3E, S15 Table). Because NHR-8 loss changed the response to dafa#3, a ligand of DAF-12, we then tested the double mutant, *nhr-8(ok186); daf-12(rh61;rh411)*. We found that Pdda is strongly increased in *nhr-8; daf-12* double mutant worms, to a similar extent as in ascaroside-deficient *daf-22* worms (Fig 3F and 3G, S7 Fig, S15 Table). Addition of dafa#3 did not change Pdda in *nhr-8;daf-12* worms, consistent with the notion that DAs act via binding to DAF-12 (Fig 3F, S8 Fig, S15 Table).

The strong Pdda observed for *nhr-8;daf-12* double mutant worms further provided the opportunity to corroborate that accumulation of worm-deposited chemical signals underlie this phenotype. For this purpose we compared time points of first egg laying of HD and ISO *nhr-8;daf-12* double mutant worms with the time point of first egg laying of ISO *nhr-8;daf-12* double mutant worms raised on plates pre-incubated with *daf-22* worms. We found that ISO *nhr-8;daf-12* double mutant worms raised on *daf-22*-pre-incubated plates developed as fast as HD *nhr-8 daf-12* double mutant worms (Fig 3H, S6 Table). This result confirms that Pdda is due to accumulation of a worm-deposited chemical signal and that this signal is responsible for the enhanced Pdda phenotype of both peroxisomal β-oxidation mutant and *nhr-8;daf-12* double mutant worms. Taken together, our results show that NHR-8 plays a central role for Pdda, and that although DAF-12 is only partially required for Pdda, developmental timing is affected by DAs in an NHR-8- and DAF-12-dependent manner.

### Larval population density predetermines adult lifespan

**Fig 4:**
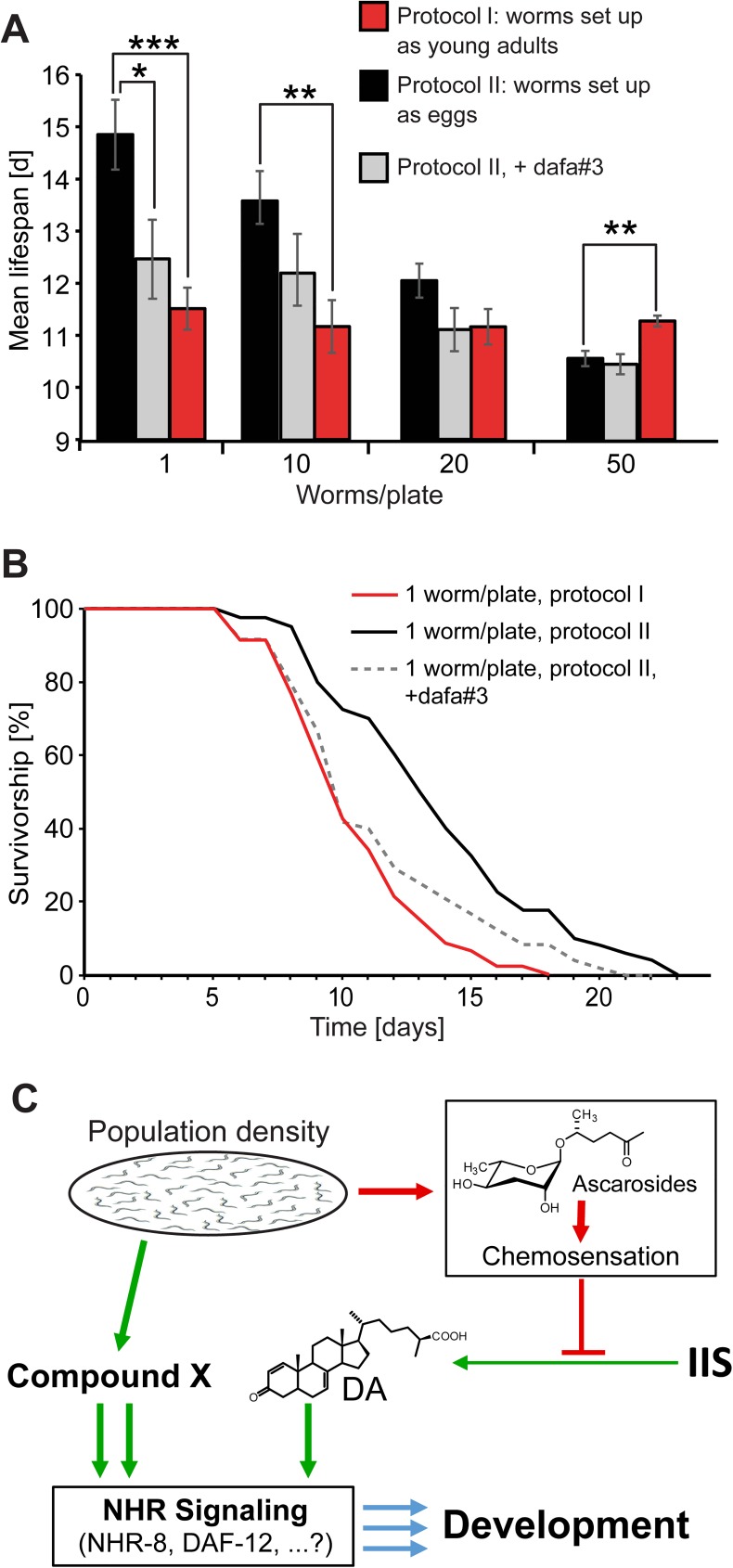
Population density affects lifespan of *C*. *elegans* hermaphrodites and a model for Pdda. **A)** Mean lifespan of hermaphrodites at different population densities, set up as eggs on mock treated plates (black bars) or set up as eggs on plates containing 100 nM dafa#3 (grey bars), or raised on plates of >100 animals and moved onto mock-treated assay plates at indicated densities at 62 h (red bars). **B)** Aging curves for hermaphrodites kept at 1 worm/plate, set up by isolating worms from plates with >100 hermaphrodites at 62 h (protocol I, red) or as eggs (protocol II, black [control] and grey dashed [with 100 nM dafa#3]). Error bars, SEM; ***P < 0.0001; **P < 0.001; *P < 0.05. **C)** Model for the roles of small molecule signaling in Pdda. Population density controls development via two competing small-molecule cascades. High population density results in accumulation of ascarosides that downregulate DA biosynthesis and thereby retard developmental progression. This effect is antagonized by a second chemical signal, here named compound X, that accelerates development and represents the proximal cause of Pdda.

Previous studies demonstrated that population density affects *C*. *elegans* lifespan in a sex-specific manner. Lifespan of *C*. *elegans* males starkly decreases at high worm densities, whereas population density did not have a significant effect on the lifespan of hermaphrodites [12]. In addition, the presence of male worms has been shown to starkly accelerate aging in hermaphrodite worms (male-induced demise, MID [13]). Based on our finding that population density affects the rate of larval development, we decided to re-investigate the effect of population density on hermaphrodites. For this purpose, we used two different experimental setups. In protocol I, we transferred hermaphrodite worms from high-density plates at the young adult stage onto the experimental plates, at densities of 1, 10, 20, and 50 worms per plate. This setup closely mimics the experimental design of the earlier study of Gems and Riddle [12] and most published aging studies. In protocol II, we set up plates with 1, 10, 20, and 50 eggs, and worms were kept at these population densities throughout development and adulthood. Under protocol I, population density did not significantly affect adult lifespan (Fig 4A, S16, S17, S18, S19 and S20 Tables), in agreement with earlier results [12]. In contrast, for worms set up under protocol II, mean adult lifespan was negatively correlated with population density. For example, the mean lifespan of isolated worms was about 5 days longer than that of worms that were raised in groups of 50 worms (Fig 4A, S8 Fig, S16, S17, S18, S19 and S20 Tables). Comparing the lifespan of isolated worms (1 worm/plate), worms raised in isolation from hatching lived almost 30% longer than worms raised on high density plates before isolation at the young adult stage. These results indicate that population density experienced during larval development contributes significantly to determining hermaphrodite lifespan.

Next we tested the effect of added dafa#3 on the adult lifespan of hermaphrodites at different population densities. Previous studies indicate that added DAs do not directly affect the adult lifespan of hermaphrodites [31]. In contrast, we found that dafa#3 significantly decreased the median lifespan of hermaphrodites at low population densities, when set up as eggs. Whereas the lifespan of hermaphrodites raised at a density of 20 or more worms per plate was similar to that of mock treated controls, lifespan of isolated worms on dafa#3-treated plates was about 20% shorter than for mock-treated worms (Fig 4A and 4B, S17 Table). This indicates that larval exposure to dafa#3 reduces adult lifespan of isolated worms. These results do not directly contradict earlier studies of the effects of DAs on adult lifespan [31], since these studies were performed with 20–40 worms per plate. At these worm densities, accumulation of other signals, for example ascarosides or the chemical signals that underlie Pdda, may mask the lifespan-shortening effect of DAs. Larval exposure to dafa#3 thus counteracts the effects of population density on both larval development and adult lifespan.

## Discussion

The nematode *C*. *elegans* is an important model for the study of metazoan development and aging. However, the effects of population density on developmental progression and adult lifespan have not been studied extensively, except for conditions that induce developmental arrest at the L1 or dauer stage. Our results demonstrate that *C*. *elegans* hermaphrodite development is regulated by larval population density. The finding that developmental acceleration at high population densities is starkly enhanced in ascaroside-deficient peroxisomal β-oxidation and chemosensory mutants indicates that in wildtype worms, Pdda is partially suppressed or antagonized by population density-dependent accumulation of dauer-inducing ascarosides (for a simplified model, see Fig 4C). We further show that Pdda is due to population density-dependent accumulation of a worm-deposited chemical signal. Therefore, it appears that *C*. *elegans* development under non-dauer inducing conditions is regulated by a push-pull mechanism based on at least two different types of pheromone-like signals: (i) chemosensation of ascarosides, which slows down development, possibly via the same pathways known to induce dauer entry by downregulating steroid hormone (DA) biosynthesis, including the *daf-2* insulin/insulin-like growth factor and the *daf-7* TGF-β signaling pathways [9], and (ii) a second pheromone-like chemical signal, named compound X in Fig 4C, that accelerates development and whose population density-dependent accumulation is the proximal cause of Pdda.

In contrast to ascarosides, compound X does not appear to be perceived via the cilia of sensory neurons, given that cilia-defective *osm-6* mutants, which do not respond to ascarosides, show increased Pdda, similar to the enhanced Pdda phenotype in ascaroside-defective *daf-22* or *dhs-28* mutants (Fig 2). Abolishment of Pdda in both *daf-2*/insulin receptor and *daf-16*/FOXO mutants argues that insulin/IGF signaling plays a complex role for Pdda, as could be expected given that both dauer-inducing ascarosides and dafachronic acids reduce Pdda (Figs 2D and 3D–3F, S6 Fig) and mutations of *daf-2* and *daf-16* affect both DA production [34,36] and sensitivity to ascarosides [8,9]. Furthermore, developmental acceleration by compound X may interact with the insulin/IGF signaling pathway via DA- and ascaroside-independent mechanisms.

Significantly, our results indicate that the effects of the Pdda signal on development involve two nuclear hormone receptors, DAF-12, which has been studied in great detail as the master switch between dauer arrest and reproductive development[9,39], and the much less well characterized NHR-8. Like most nuclear receptors in *C*. *elegans* and many NHRs in higher animals, NHR-8 remains to be de-orphaned, that is, no endogenous ligands have been identified. However, NHR-8 has been shown to be involved in cholesterol and DA homeostasis [38] and, more recently, this nuclear receptor been shown to play a role for dietary restriction-mediated longevity [37]. Notably, NHR-8 was shown to act downstream of DA biosynthesis in this pathway, even though there is no evidence that DAs activate or directly bind to this nuclear receptor. Our observation that Pdda is starkly enhanced in the *nhr-8; daf-12* double mutant indicates that the pheromone-like signal compound X does not represent a ligand of either NHR-8 or DAF-12. However, given that Pdda is fully abolished in *nhr-8* null mutants, enhanced in the *nhr-8; daf-12* double mutant, but similar to wild type in *daf-12* and *daf-9; daf-12* mutants, NHR-8 appears to play a central role for the regulation of developmental timing. Particularly curious is the finding that even though Pdda is abolished in *nhr-8* null mutants and abolished in wildtype by addition of exogenous DA, the addition of exogenous DA in *nhr-8* null mutants largely restores Pdda, that is, the effect of added DA is opposite in *nhr-8* mutants compared to wildtype. It is not known whether NHR-8 and DAF-12, like their closest mammalian homologs, e.g. the vitamin D receptor (VDR), act as heterodimers. In mammals, VDR forms a heterodimer with the retinoid X receptor (RXR), and binding of their respective endogenous ligands to either VDR or RXR directly affects recruitment of transcriptional coregulators by the unliganded partner receptor [40]. Correspondingly, it seems possible that Pdda is mediated in part via direct interactions of NHR-8 and DAF-12 within a heterodimer. Clarification of the mechanisms by which NHR-8 regulates developmental timing will benefit from identification of endogenous ligands of this NHR, in addition to identification of compound X as the primary cause of Pdda. Taken together, our results show that the role of nuclear hormone receptors for regulating developmental timing extends beyond DAF-12 and the dafachronic acid signalling cascade.

Inspired by earlier work on the effects of the population density of *C*. *elegans* males on male [12] and hermaphrodite lifespan ("MID") [13], we also investigated whether population density affects adult lifespan in *C*. *elegans* hermaphrodites. We found that high larval population density, but not high population density experienced during adulthood, significantly reduces hermaphrodite lifespan. In this regard, population density-dependent shortening of hermaphrodite lifespan differs from MID, since male-derived small molecules shorten hermaphrodite lifespan through exposure during adulthood [13]. It is unclear whether Pdda and the effects of larval population density on adult lifespan derive from a common molecular mechanism, e.g. whether compound X is responsible for the reduced lifespan of hermaphrodites raised under crowded conditions, and to what extent accumulation of lifespan-extending ascarosides, e.g. ascr#2 and ascr#3 [17], antagonize hermaphrodite progeria on high density plates. However, our lifespan assays with added DA demonstrate that larval population density not only regulates hermaphrodite adult lifespan, but also affects responses to added small molecules, suggesting that responses to other environmental stimuli or genetic interventions may be affected as well. Therefore, differences in larval population densities prior to set-up of the actual aging assays, generally at the young adult stage, provides a potential explanation for conflicting findings about the roles of specific environmental and genetic factors on lifespan in *C*. *elegans*.

Our work further shows that measuring onset of egg laying as a marker for full maturity represents an effective means to investigate the effect of environmental or genetic changes on development. Developmental maturation in animals is highly plastic, and a great variety of environmental factors have been shown to affect rates of development [9], although the underlying mechanisms are unclear. In humans, the onset of sexual maturation has continued to trend toward younger ages, likely as the result of yet unidentified environmental stimuli [41,42]. Elucidation of the signaling pathways mediating Pdda in *C*. *elegans* may provide insights in the control of developmental timing in higher animals, including humans.

## Materials and methods

### 1. Nematode and bacterial strains

Unless indicated otherwise, worms were maintained on Nematode Growth Medium (NGM) 6 cm diameter Petri dish plates with *E*. *coli* OP50 (http://www.wormbook.org/methods, Brenner 1974). The following *C*. *elegans* strains were used: wild type Bristol N2, *daf-16(mu86)*, *daf-16(m26)*, *daf-2(e1368)*, *daf-2(e1370)*, *daf-12(rh61 rh411)*, *ins-11(tm1053)*, *daf-22(ok693)*, *daf-22(m130)*, *dhs-28(hj8)*, *dhs-28(2581)*, *daf-9(rh50)*, *daf-9(dh6); daf-12(rh61 rh411)*, *sir-2*.*1(ok434)*, *odr-3(n2150)*, *tax-4(ks28)*, *osm-6 (p811)*, *egl-4(n477)*, *nhr-49(nr2041)*, *hsf-1(sy441)*, *nhr-8(ok186)*, *nhr-8(tm1800)*, *nhr-8(ok186);daf-12 (rh61 rh411)*, *mec-4(e1399)*.

### 2. Population density dependent egg laying (Pdda) assays

#### 2.1. Protocol A

50–100 pre-synchronized well fed adult gravid *C*. *elegans* hermaphrodites were placed on fresh 6 cm NGM plates seeded with OP50 *E*. *coli* bacteria (100 μL fresh bacteria were spread on the center of the plate and grown overnight at room temperature to produce a bacterial lawn of approximately 2–3 cm diameter.) and were allowed to lay eggs for 1 hour. After removal of the adults, eggs were transferred using a worm pick onto fresh plates at 1 egg/plate, 10 eggs/plate, and 41 eggs/plate (35–50 eggs per plate, average of 41, over 16 assays). For assays with 242 (190–294, 14 assays) and 909 (750–980, 5 assays) eggs per plate, gravid adults were treated with hypochlorite, and eggs were placed onto plates using a wormsorter (cytometry-based object parametric analysis and sorting system, COPAS Biosort, Union Biometra, Belgium). The time point of egg transfer onto plates was defined as start time of the experiment. Starting at 60 h after setup, plates were scored continuously every hour for laying of the first egg. Plates were incubated at 20°C until scoring at room temperature (~21°C).

#### 2.2. Protocol B

For synchronisation, 50–100 worms were allowed to lay eggs for 1 h on NGM plates seeded with OP50 *E*. *coli* bacteria, then adult worms were removed and the resulting F1 generation was grown for 72 h at 20°C. From those plates, 50–100 worms were transferred to a fresh NGM plate (100 μL fresh bacteria were spread onto the center of a plate and grown overnight at 23°C to produce a bacterial lawn of approximately 2 cm diameter). Worms were allowed to lay eggs for 1 h and the desired number of eggs - 1 (ISO worms) or 50–100 eggs per plate (High Density (HD) worms) - were transferred using a worm pick to fresh plates. The time point of egg transfer was defined as start time of the experiment. After 59 h at 20°C, 10–30 worms from each condition were transferred individually onto fresh 6 cm NGM plates seeded with OP50 *E*. *coli* bacteria. Usually, 15–25 ISO plates and 3 HD plates were set up for each experiment. Animals were scored for egg laying at 60 h after birth and then every hour. Embryonic survival was assessed prior to scoring. Generally, survival was 95% or greater, on both HD and ISO plates. Assays in which larger numbers of eggs did not hatch, on either the ISO or HD arms, were not scored. All assays were repeated at least three times, unless noted otherwise in the Supporting Tables.

#### 2.3. Staging of developing worms

For synchronisation, 50–100 wildtype worms were allowed to lay eggs for 1 h on NGM plates seeded with OP50 *E*. *coli* bacteria, then adult worms were removed and the resulting F1 generation was grown for 72 h at 20°C. From those plates, the desired number of eggs—one or ~100 per plate—were transferred to fresh seeded plates. After 52 hours, 60 single worms per condition were examined microscopically for their developmental stage. Staging was based on vulval shape and the degree of reflection of the gonad.

#### 2.4. Mutant analysis

Mutant animals and wildtype (N2) controls were synchronized and isolated according to protocol B and scored for 1^st^ egg laying starting at 60 h. For comparing Pdda in mutant and wildtype worms, the difference between the time points of first egg laying for mutant worms under ISO and HD conditions was measured and then normalized relative to the average time to first egg laying in this mutant. The size of the effect was then expressed in percent of Pdda observed in the parallel wildtype experiment. Data shown are average of at least three independent biological replicates. Animals were scored using a Leica DM 5500B microscope and a Q Imaging 200R camera.

#### 2.5. Assays with synthetic ascarosides and Das

To test for effects of ascarosides and DAs on Pdda, 6 cm NGM plates containing 400 nM of synthetic ascarosides (ascrs) or 100 nM of synthetic dafachronic acids were prepared as follows (compounds were synthesized as described [32,43,44]). For preparation of ascaroside-containing NGM plates, samples of ascr#2 or ascr#3 were dissolved in 100% ethanol producing 2-10 mM stock solutions, which were diluted further with water to yield solutions of 20 μM. These aqueous solutions were prepared freshly prior to each experiment. For preparation of DA-containing NGM plates, dafa#1, dafa#2, or dafa#3 were dissolved in 100% ethanol to produce stock solutions of 5 μM. Control solutions for mock treatment had corresponding amounts of ethanol added. Onto the surface of each experimental NGM plate, 200 μL of ascaroside, DA, or mock solution was evenly spread. Plates were then allowed to dry for 24 h at 20°C. Subsequently, 60 μL of bacteria (*E*. *coli* OP50 pellet), freshly grown at 37°C in LB media, was spread onto the plates. Plates were then incubated for 24 h at 20°C. All assays using ascarosides or DAs were conducted as described above for "protocol B", thus exposure to the tested compounds started with the transfer at the egg/embryo stage. Worms were isolated to fresh compound- or mock-treated NGM plates at 59 h and scored for 1^st^ egg laying starting at 60 h. The change in Pdda was measured in percent of Pdda observed for untreated worms, as described above. Data shown are average of at least three independent biological replicates, unless noted otherwise.

### 3. Pre-incubation assays

Wildtype (N2) or *daf-22(ok693)* mutants worms were synchronized by letting 50–100 young adults lay eggs for 1 h on each of 20–30 6 cm NGM plates with *E*. *coli* OP50 bacteria. This resulted in plates with 100–150 wildtype or *daf-22* eggs per plate. Worms were allowed to grow until reaching larval stage 4, before egg laying started. At this time all worms were carefully picked off the plates. These wildtype- or *daf-22*-preincubated plates were then used in subsequent Pdda assays with wildtype (using wildtype-preincubated plates), *daf-22* mutant (using *daf-22*-preincubated plates) or *nhr-8;daf-12* double mutant (using *daf-22*-preincubated plates) worms. For these assays, wildtype, *daf-22(ok693)* mutant, or *nhr-8(ok186); daf-12(rh61 rh411)* double mutant worms were synchronized by egg laying of 50–100 young adults for 1 hour on three 6 cm-NGM plates. Freshly laid single eggs were either transferred to pre-incubated 6 cm NGM plates ("HD-preincubated ISO", 20–30 plates per assay) or to untreated OP50 coated NGM plates ("ISO", 20–30 plates per assay). Simultaneously, cohorts of 50–60 freshly laid eggs were transferred to OP50 coated 6 cm NGM plates ("HD", 3 plates per assay). Single worms from the ISO, HD-preincubated ISO, and HD plates were isolated to fresh NGM plates (20–30 plates per condition) at 59 h. Starting at 60 h, worms were scored every hour for laying of the 1^st^ egg.

### 4. Lifespan assays

50–100 hermaphrodite N2 worms from synchronized plates were placed onto NGM plates with fresh *E*. *coli* OP50 bacteria and allowed to lay eggs for 1 h. **Protocol I.** From plates with freshly synchronized eggs, 100 eggs were transferred to fresh plates seeded with *E*. *coli* OP50 bacteria. At young adult stage, 62 h after birth, worms were transferred to fresh plates in groups of 1, 10, 20, and 50 worms per plate. **Protocol II.** From plates with freshly synchronized eggs, 1, 10, 20, and 50 eggs were transferred to fresh plates seeded with *E*. *coli* OP50 bacteria. For each assay, 20–30 plates with 1 worm, 5–10 plates with 10 worms, and 3–5 plates with 20 or 50 worms were set up. For ageing studies with dafachronic acids, worms were set up on dafa#3 containing plates prepared as described above according to protocol II. Worms were transferred daily onto fresh plates until cessation of egg laying (~ day 8) and every 3 days after that until the experiment was completed. Animals were scored as dead if they failed to respond to a tip on the head and tail with a platinum wire. Worms with internal hatching, exploders or animals that crawled off the plate were excluded. Aging experiments were conducted in two different laboratories, in Kiel, Germany, and Ithaca, NY, as indicated in the Supporting Tables.

### 5. Statistics

The Pdda metric was calculated from time differences of mean times to first egg laying ("t"), as follows: Pdda [%] = [t(ISO_mutant_)-t(HD_mutant_)]/[t(ISO_wildtype_)-t(HD_wildtype_)]*t(ISO_wildtype_)/t(ISO_mutant_)*100. Whereas absolute developmental times exhibit a high degree of variability, acceleration of development under HD conditions as measured by this Pdda metric is much less variable (see Supporting Tables and S10 Fig). The standard deviation of the difference in times between onset of egg laying under isolated versus high density conditions was calculated: STD_time difference_ = sqrt(STD_iso_^2/N_iso_+STD_HD_^2/N_HD_); P-values were calculated using t-tests, and the Bonferroni correction was used to assess significance for multiple comparisons. Box plots were generated with the “R-Studio” software. Lifespan data were analysed using the SPSS software for determination of mean and median lifespan, generation of life span curves and log-rank tests.

**Supplementary Information** is linked to the online version of the paper at http://journals.plos.org/plosgenetics

## Supporting information

S1 Fig Pdda and development of *C*. *elegans* wild type larvae. **A-E** Micrographs of worms at different time points during development, grown at isolation (ISO, upper panels) or at High Density (HD, lower panels). **A)** 22 hours after plating of synchronized eggs, larval stage 1. **B)** 38 hours (larval stage 3, bilateral extension of gonadal arms); **C)** At 52 hours, ISO worms are at stage L4.2, whereas HD worms are at stage L4.9. **D)** At 57 hours, ISO worms are stage L4.5 whereas HD worms are at Young Adult (YA) stage. Black and white asterisks indicate the position of the vulva; black arrows indicate the position of one of the distal tip cells of the gonad, magnification: 63x. **E)** Zoomed-in images of the vulval region of 22, 52, and 57 hour old larvae grown under ISO (upper panels) and HD (lower panels) conditions, red bar indicates 25 μM. **F)** Pdda assay with animals isolated at different time points from synchronized plates with >100 worms, three assays per condition with 22–25 isolated animals. Error bars, STD, **P < 0.001, for full data see S2 Table.
(TIF)

S2 Fig Population-density dependence of development. **A)** Number of offspring on day 1 of adulthood of worms raised in isolation (1 worm/plate) or at densities of 10 and 100 worms/plate. Worms were raised at 1, 10, or 100 worms/plate until 58 h and then worms from all groups were transferred onto new plates at 1 worm/plate. Number of offspring was counted at 84 h. **B)** Embryo volume of offspring from worms raised at different densities. **C)** Lifetime fecundity of worms raised at different densities. **D)** Time course of egg laying of worms raised at different densities. Error bars, SEM, ***P < 0.0001; **P < 0.001; *P < 0.05.
(TIF)

S3 Fig Pdda in *nhr-49(nr20141)*, *hsf-1(sy441)* and *sir-2*.*1(ok434)* mutant strains. Error bars: STD. For full data see S4 Table.
(TIF)

S4 Fig The ascaroside ascr#2 suppresses Pdda in wildtype but not in chemosensory mutants *tax-4(ks28)* and *osm-6(p811)*. Error bars, STD; ***P < 0.0001; **P < 0.001; *P < 0.05. For full data see S9 Table.
(TIF)

S5 Fig Pre-conditioning Pdda *assay*. Time point of first egg laying of wildtype (N2) worms on HD and ISO plates, as well as on plates pre-incubated plates with wildtype (N2) worms at high density. Box plot showing medians, upper and lower quartiles, whiskers at 1.5 IQR; error bars, SEM; *P < 0.05. For full data, see S6 Table.
(TIF)

S6 Fig Effect of Dafachronic Acids (DAs) on Pdda in *daf-22* mutant worms. **A)** Pdda in *daf-22(ok693)* mutant worms treated with different DAs at 100 nM; DA-mix: mixture of dafa#1, dafa#2, and dafa#3 at 100 nM each, error bars, STD; ***P < 0.0001; **P < 0.001; *P < 0.05. **B)** Time point of 1^st^ egg laying in *daf-22(ok693)* mutant worms treated with indicated concentrations of dafa#3; error bars, STD; ***P < 0.0001; **P < 0.001; *P < 0.05. For full data, see S14 Table.
(TIF)

S7 Fig Population density dependent effects on development in *daf-22* and *nhr-8;daf-12* mutants. Images show *daf-22(ok693)* (**A**) and *nhr-8(ok186);daf-12(rh61rh411)* (**B)** mutant worms grown in isolation (ISO, upper panel) or in groups of >50 animals per plate (HD, lower panels). Red arrows and red lines indicate the position of the developing gonad, white arrows indicate the distal tip cell of the gonad (DTC), white numbers refer to sub-stages of larval stage 4, characterized by shape of the vulva and degree of gonadal reflection.
(TIF)

S8 Fig Population density and lifespan. **A)** Survival curves (generated in SPSS) of 67, 13, and 1 worms/plate set up as eggs (Protocol B), without ethanol on the plates (ethanol was used for the lifespan data presented in Fig 4 and S9 Fig). **B)** Mean lifespan of worms in **A**. Error bars, SEM; ***P < 0.0001; **P < 0.001; *P < 0.05. For full data, see S16 Table.
(TIF)

S9 Fig Population density and lifespan. Survival curves (generated in SPSS, mean lifespans shown in Fig 4A) of **A)** worms set up as Young Adults at densities of 1, 10, 20, and 50 worms per plate (wpp); **B)** worms set up as eggs; **C)** worms set up as eggs using 100 nM dafa#3. Plates used for the experiments in **A** and **B** were mock-treated with the same amount of ethanol used in the preparation of dafa#3-containing plates in **C**. For full data, see S17 Table.
(TIF)

S10 Fig Example data sets for developmental timing differences on ISO and HD plates of WT worms compared to *daf-22* mutants. **A)** Timepoint of first egg laying (hours) in four separate experiments run at different times from 2014–2016; ISO: isolated as eggs; HD: grown at high density, isolated after 59 hours; **B)** Same data represented in terms of the Pdda metric; red line indicates 100% of wildtype Pdda. Error bars: STD, ***P < 0.0001; **P < 0.001; *P < 0.05.
(TIF)

S1 Table Population density dependent egg laying in *C*. *elegans* wild type (N2). Comparison of protocols A and B (data shown in Fig 1A and 1C). ISO: isolation (1 worm per plate), HD; high density, wpp = worms per plate. ^a^ p-values for comparison of 10 wpp, 41 wpp, 242 wpp, 909 wpp with ISO (protocol A), and for comparison of HD_61h_ and HD_48h_ with ISO (protocol B). ^b^plated with a worm sorter (COPAS Biosort, Union Biometra, Geel, Belgium).
(DOCX)

S2 Table Population density-dependent egg laying in *C*. *elegans* wild type (N2). Developing larvae were isolated at different time points during development, indicated as hours after synchronization. Data shown in S1 Fig, part F. ^a^ p-values for comparison of ISO, 8 h; ISO, 24 h; ISO, 39 h; ISO, 54 h and ISO, 61 h with ISO, 0 h (protocol B).
(DOCX)

S3 Table Developmental stages of *C*. *elegans* wild type (N2) worms at different population densities at 52 h (data shown in Figure panel 1D). (DOCX)

S4 Table Population density dependent egg laying in *C*. *elegans* mutant strains using protocol B. Data are shown in Fig 1E and S3 Fig. ISO: isolation (1 worm per plate), HD; high density (50–100 worms per plate). ^b^Pdda assays with *nhr-49(nr2041)*, *hsf-1(sy441)* and *sir-2*.*1(ok434)* mutants were performed only once, with >12 plates each for ISO and HD conditions.
(DOCX)

S5 Table Population density dependent egg laying in mutant strains with defects in peroxisomal β oxidation using protocol B. Data shown in Fig 2A. ISO: isolation (1 worm per plate), HD; high density (50–100 worms per plate).
(DOCX)

S6 Table Pre-incubation assays. Timepoint of 1^st^ egg in N2 wildtype, *daf-22(ok693)* and in *nhr-8(ok186) daf-12(rh61411)* mutants grown on regular plates or on plates pre-populated with *daf-22(ok693)* or N2 wildtype larvae (data shown in Figs 2G, 3H, and S5 Fig). ISO: isolation (1 worm per plate), HD; high density (50–100 worms per plate); HDpreISO: assay with one worm per plate, using HD-pre-conditioned plates. Assays with *daf-22(ok693)*: two independent biological repeats with >20 plates per condition; assays with N2 and *nhr-8(ok186); daf-12(rh61rh411)*: one assay each with >20 plates per condition.
(DOCX)

S7 Table Population density dependent egg laying in wild type (N2) and *daf-22(ok693)* mutant strains treated with 100 nM of ascr#2 or ascr#3. Data are shown in Fig 2D and 2E. ISO: isolation (1 worm per plate), HD; high density (50–100 worms per plate). ^a^Assays with *daf-22(ok693)* and ascr#3: two independent biological repeats, each using >12 plates per assay, assays with *daf-22(ok693)* ascr#2: one data set, using >12 plates per condition. ^e^Ethanol containing plates (0.002% v/v)
(DOCX)

S8 Table Population density dependent egg laying in mutant strains with defects in chemosensation, using protocol B. Data are shown in Fig 2F. ISO: isolation (1 worm per plate), HD; high density (50–100 worms per plate).
(DOCX)

S9 Table Population density dependent egg laying in *tax-4* and *osm-6* mutant mutants. Assays were run using protocol B using plates containing 400 nM ascr#2, ascr#3, ascr#10 or mock treatment (data shown in S4 Fig). ISO: isolation (1 worm per plate), HD; high density (50–100 worms per plate). ^e^Ethanol containing plates (0.002% v/v).
(DOCX)

S10 Table Population density dependent egg laying in insulin pathway mutants strains. Assays were run using protocol B (data shown in Fig 3B). ISO: isolation (1 worm per plate), HD; high density (50–100 worms per plate).
(DOCX)

S11 Table Population density dependent egg laying in several different *daf-2* mutant strains using protocol B. ISO: isolation (1 worm per plate), HD; high density (50–100 worms per plate).
(DOCX)

S12 Table Population density dependent egg laying in nuclear hormone receptor signaling mutants. Assays were run using protocol B (data shown in Fig 3C). ISO: isolation (1 worm per plate), HD; high density (50–100 worms per plate).
(DOCX)

S13 Table Population density dependent egg laying in three different *nhr-8* mutant strains. Assays were run using protocol B. ISO: isolation (1 worm per plate), HD; high density (50–100 worms per plate). *same control as in S11 Table* (*daf-2(e1370*)); **same control as in S11 Table* (*daf-2(e1368*))
(DOCX)

S14 Table Population density dependent egg laying in N2 wildtype and *daf-22* mutants. Worms were mock treated or grown in presence of dafachronic acids at 100 nM (or indicated concentrations), using protocol B. ISO: isolation (1 worm per plate), HD; high density (50–100 worms per plate). Data shown in S6 Fig. ^a^Assays with *daf-22(ok693)* and dafa#1 and dafa#2: one data set each with >12 plates per condition. ^e^Plates with EtOH (0.2% v/v).
(DOCX)

S15 Table Population density dependent egg laying in *C*. *elegans* in N2 wildtype and nuclear hormone receptor signaling mutants. Worms were treated with dafachronic acids at 100 nM (or indicated concentrations) or mock-treated controls, using protocol B. ISO: isolation (1 worm per plate), HD; high density (50–100 worms per plate). Data shown in Fig 3E and 3F. ^BF^Bonferroni correction was used to determine significance for multiple comparisons. ^e^Plates with EtOH (0.2% v/v).
(DOCX)

S16 Table Lifespan assays. **Mean and maximal lifespan of wild type (N2) worms grown at different population densities.** "ISO" refers to assays with one worm per plate (wpp). Assays were conducted in Ithaca, NY, (1^st^ data set, data shown in S8 Fig) and in Kiel, Germany (2^nd^ data set). *Longest lifespan among all ISO worms in this experiment.
(DOCX)

S17 Table Lifespan assays with wild type (N2) worms. Worms were set up on experimental plates as egg (egg, lifespan protocol II) or as young adult (YA, lifespan protocol I), with or without 100 nM dafa#3 (data shown in Fig 4A–4C). "ISO" refers to assays with one worm per plate (wpp). Assays were conducted in Ithaca, NY. All plates contained ethanol (0.2% v/v). *longest lifespan among all ISO worms in this experiment.
(DOCX)

S18 Table Analysis of lifespan data obtained for N2 worms at different population densities, set up either as young adults ("YA", lifespan protocol I), or as eggs ("egg", lifespan protocol II) by pair-wise comparison, using the log-rank test in SPSS; p < 0.05 was considered significant. In all assays plates containing EtOH (0.2% v/v) were used.
(DOCX)

S19 Table Analysis of lifespan data obtained for N2 worms at different population densities, set up on plates containing 100 nM of dafa#3, either as young adults ("YA d#3", lifespan protocol I), or as eggs ("egg d#3", lifespan protocol II) by pair-wise comparison, using the log-rank test in SPSS; p < 0.05 was considered significant. In all assays plates containing EtOH (0.2% v/v) were used.
(DOCX)

S20 Table Analysis of lifespan data obtained for N2 worms at different population densities, set up as eggs on plates containing 100 nM dafa#3 ("egg, d#3", lifespan protocol II) or plates that were mock treated ("egg M", lifespan protocol II)) by pair-wise comparison using the log-rank test in SPSS; p < 0.05 was considered significant. In all assays plates containing EtOH (0.2% v/v) were used.
(DOCX)
