# Supplementary figures and images for: Larval crowding accelerates C. elegans development and reduces lifespan
Source: PLoS Genet. 2017 Apr 10;13(4):e1006717. doi: 10.1371/journal.pgen.1006717 (PMC5402976; doi:10.1371/journal.pgen.1006717)

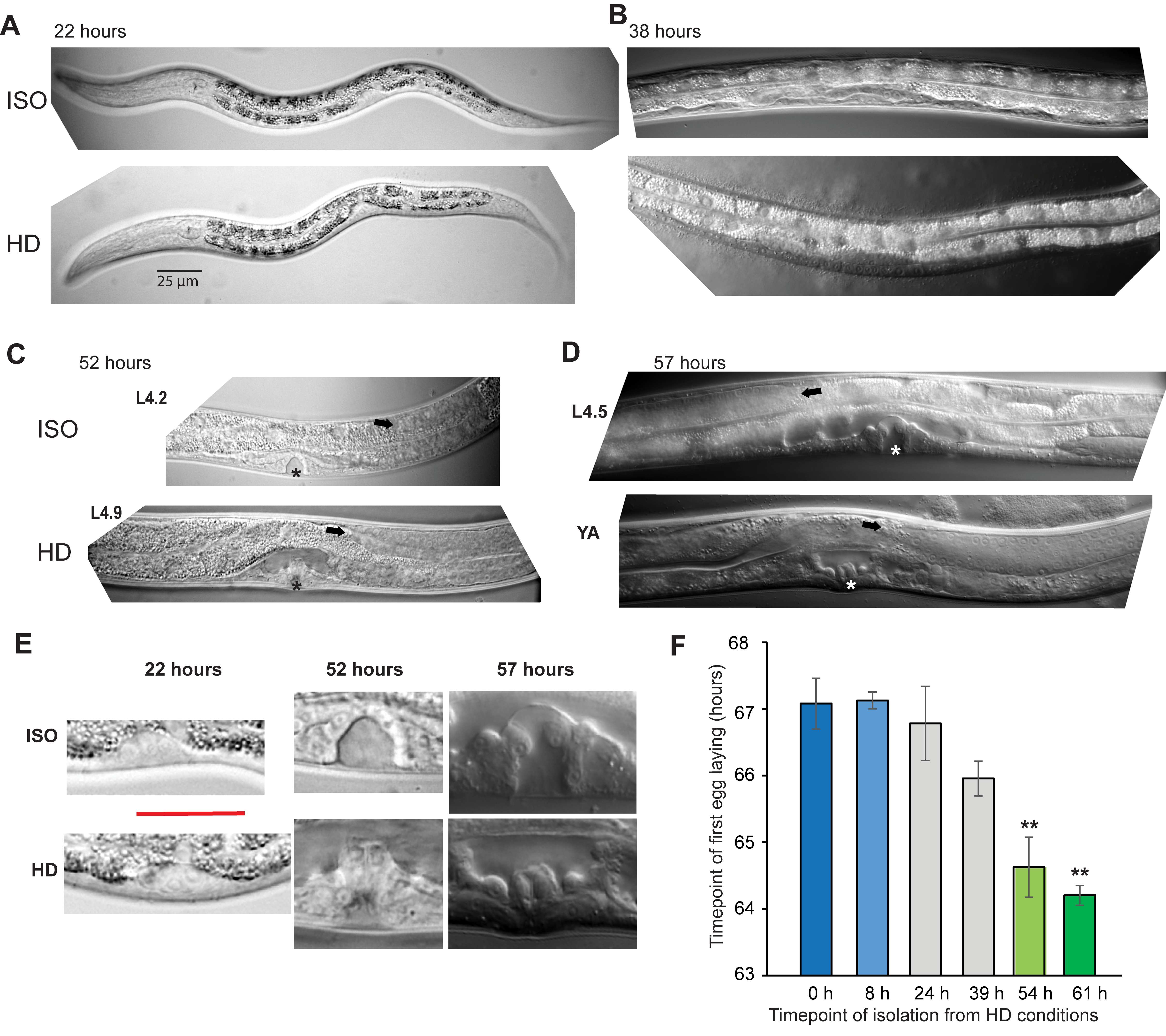

Supplement: S1 Fig — A-E Micrographs of worms at different time points during development, grown at isolation (ISO, upper panels) or at High Density (HD, lower panels). A) 22 hours after plating of synchronized eggs, larval stage 1. B) 38 hours (larval stage 3, bilateral extension of gonadal arms); C) At 52 hours, ISO worms are at stage L4.2, whereas HD worms are at stage L4.9. D) At 57 hours, ISO worms are stage L4.5 whereas HD worms are at Young Adult (YA) stage. Black and white asterisks indicate the position of the vulva; black arrows indicate the position of one of the distal tip cells of the gonad, magnification: 63x. E) Zoomed-in images of the vulval region of 22, 52, and 57 hour old larvae grown under ISO (upper panels) and HD (lower panels) conditions, red bar indicates 25 μM. F) Pdda assay with animals isolated at different time points from synchronized plates with >100 worms, three assays per condition with 22–25 isolated animals. Error bars, STD, **P < 0.001, for full data see S2 Table. (TIF) [file pgen.1006717.s001.tif]

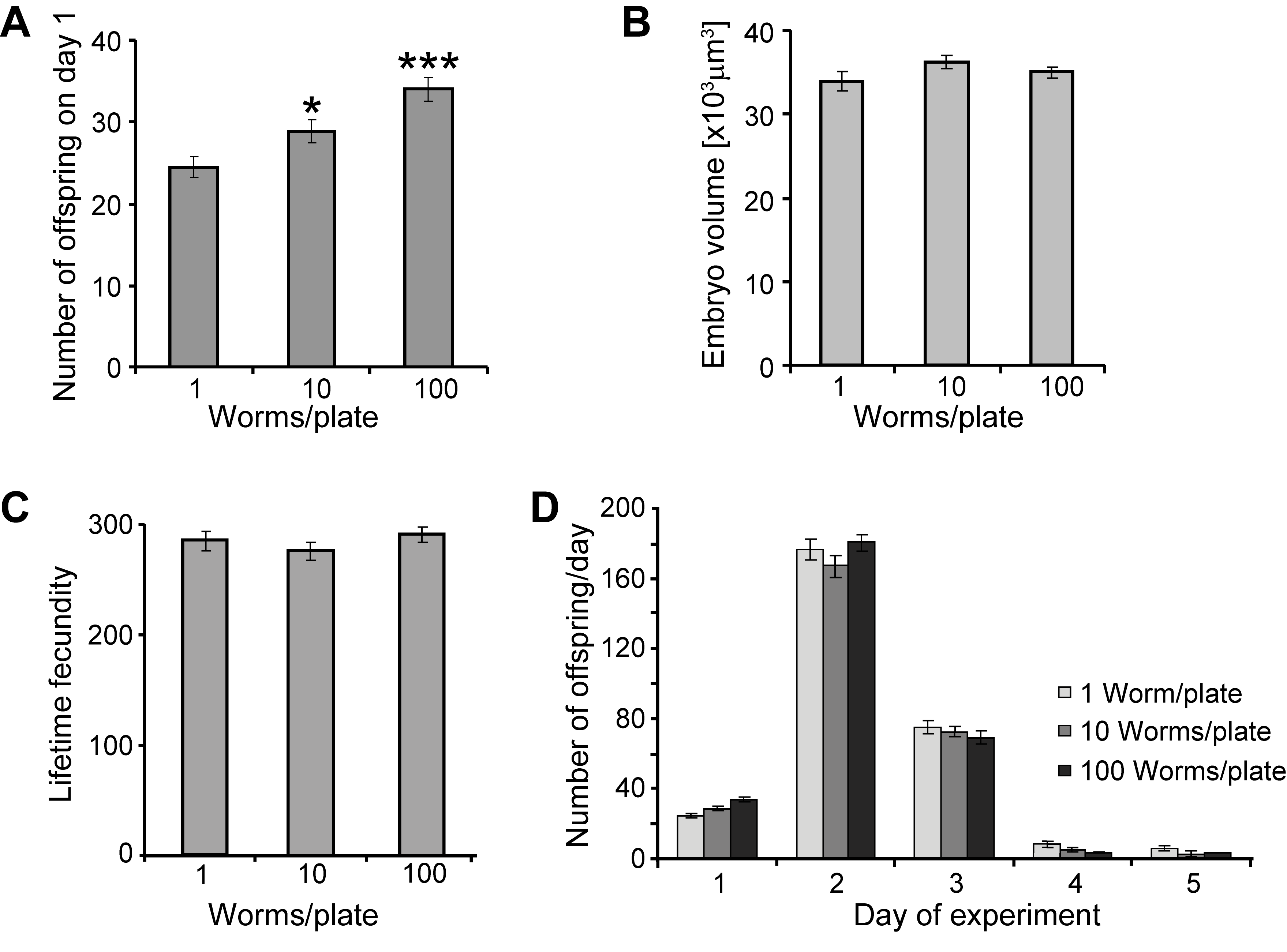

Supplement: S2 Fig — A) Number of offspring on day 1 of adulthood of worms raised in isolation (1 worm/plate) or at densities of 10 and 100 worms/plate. Worms were raised at 1, 10, or 100 worms/plate until 58 h and then worms from all groups were transferred onto new plates at 1 worm/plate. Number of offspring was counted at 84 h. B) Embryo volume of offspring from worms raised at different densities. C) Lifetime fecundity of worms raised at different densities. D) Time course of egg laying of worms raised at different densities. Error bars, SEM, ***P < 0.0001; **P < 0.001; *P < 0.05. (TIF) [file pgen.1006717.s002.tif]

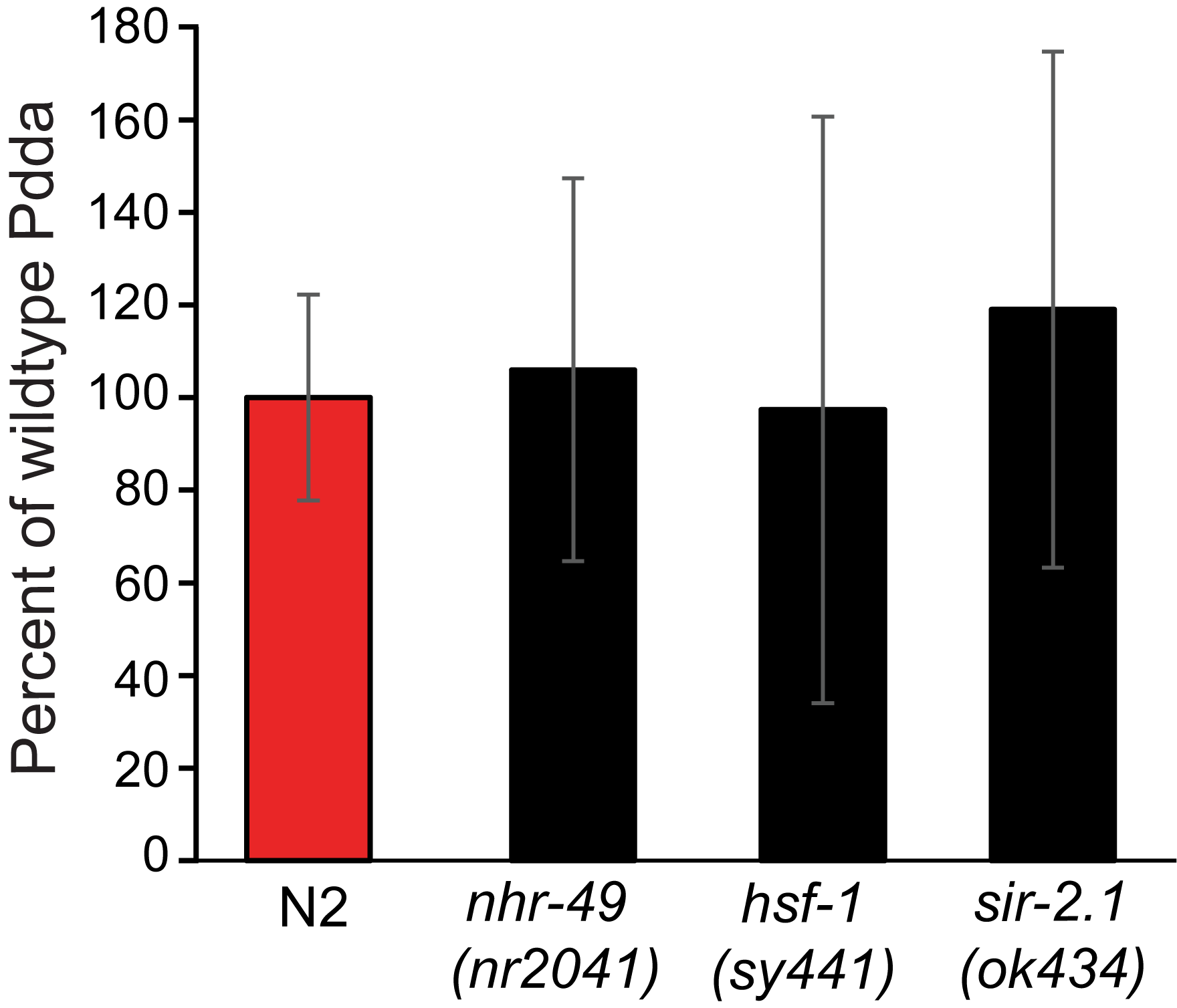

Supplement: S3 Fig — Error bars: STD. For full data see S4 Table. (TIF) [file pgen.1006717.s003.tif]

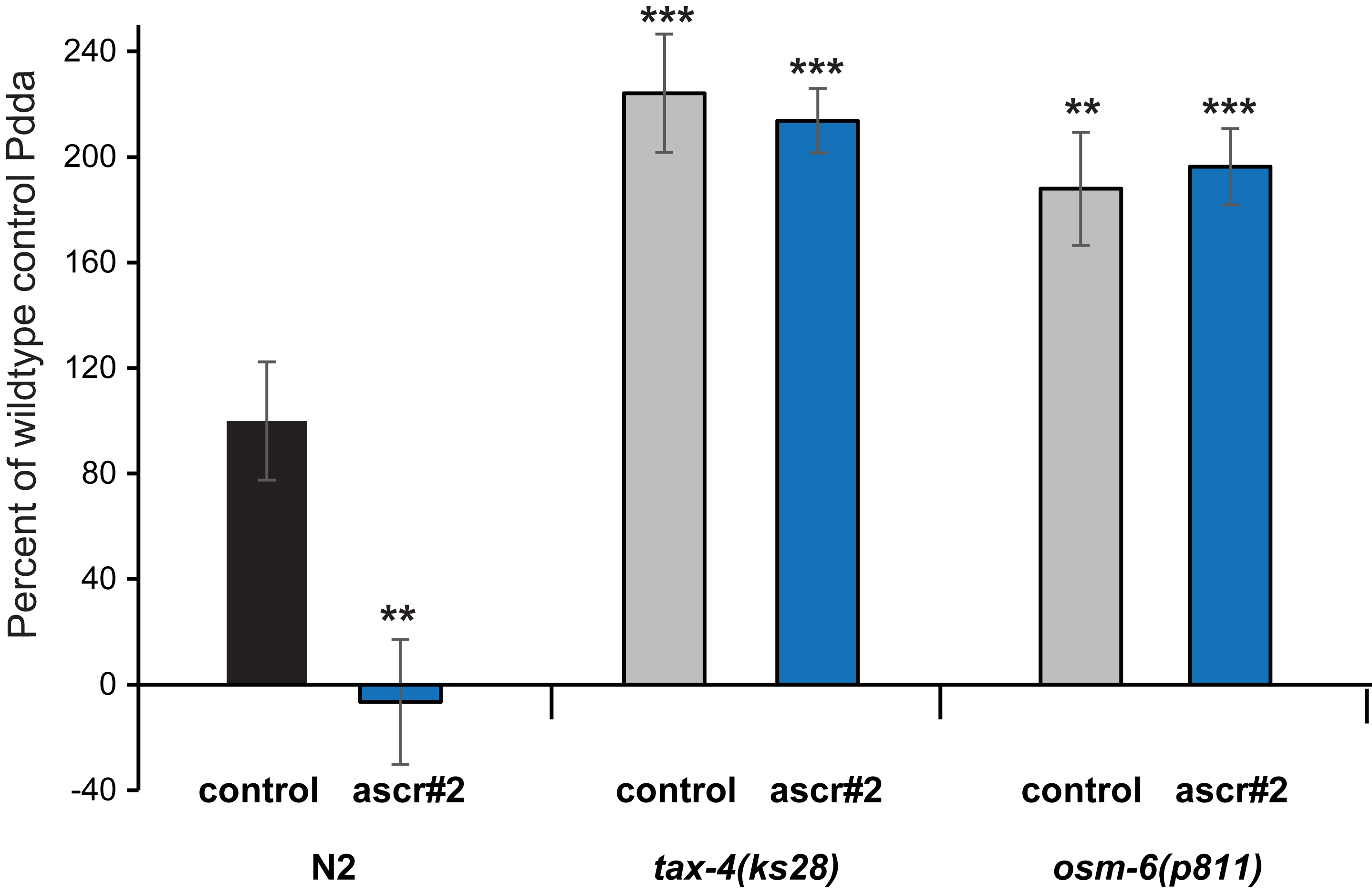

Supplement: S4 Fig — Error bars, STD; ***P < 0.0001; **P < 0.001; *P < 0.05. For full data see S9 Table. (TIF) [file pgen.1006717.s004.tif]

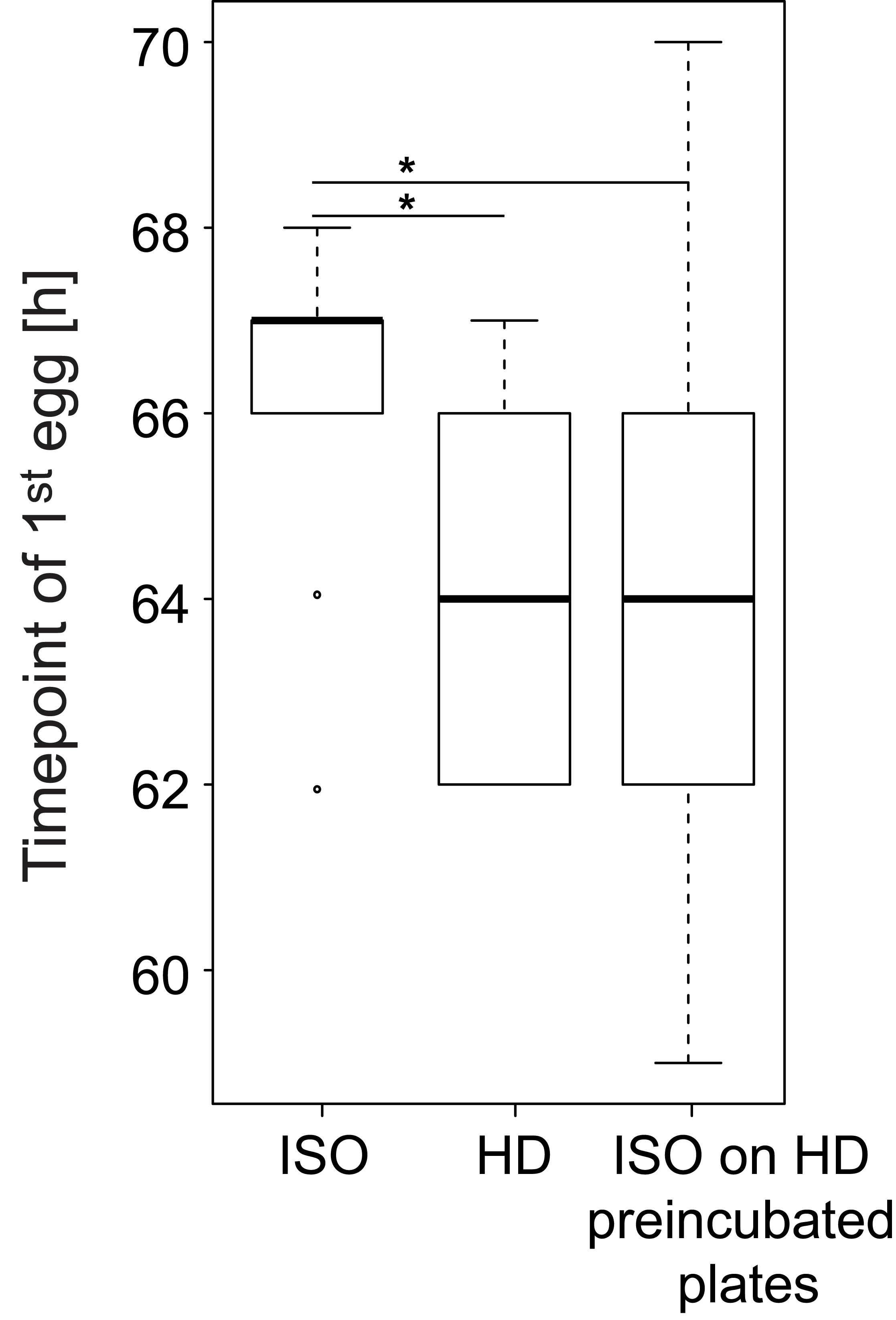

Supplement: S5 Fig — Time point of first egg laying of wildtype (N2) worms on HD and ISO plates, as well as on plates pre-incubated plates with wildtype (N2) worms at high density. Box plot showing medians, upper and lower quartiles, whiskers at 1.5 IQR; error bars, SEM; *P < 0.05. For full data, see S6 Table. (TIF) [file pgen.1006717.s005.tif]

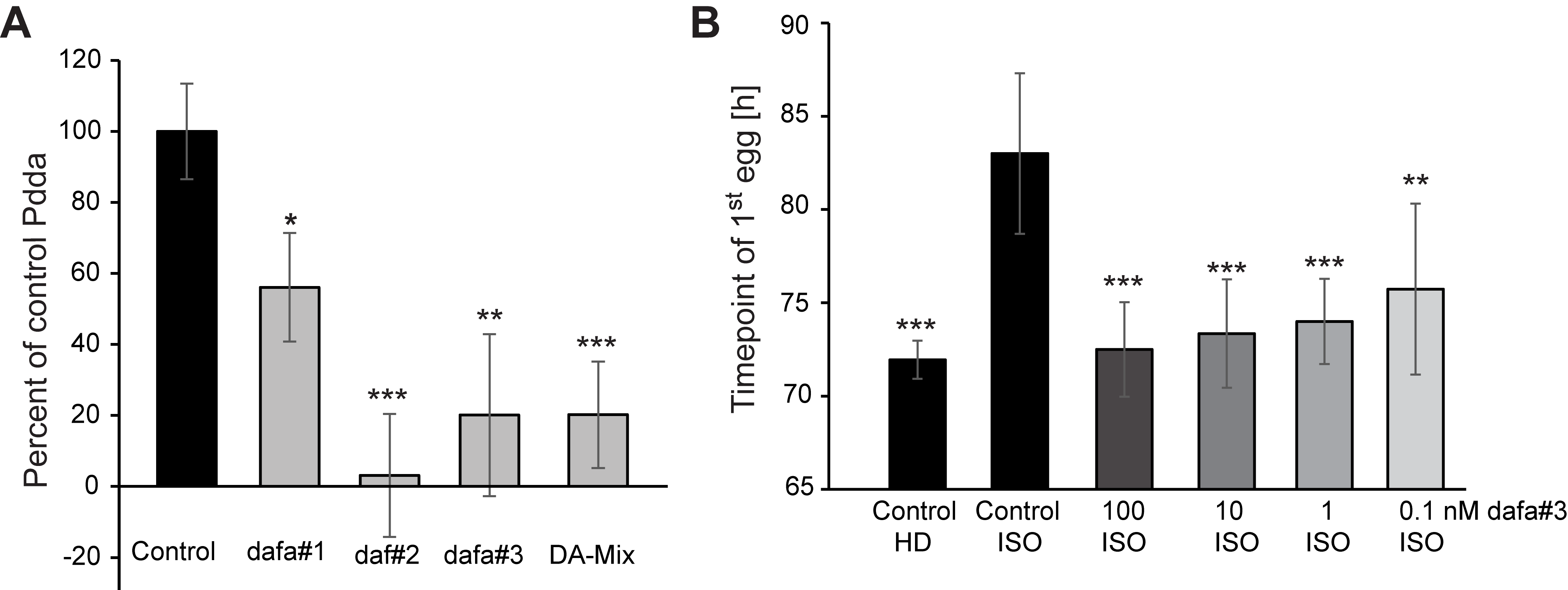

Supplement: S6 Fig — A) Pdda in daf-22(ok693) mutant worms treated with different DAs at 100 nM; DA-mix: mixture of dafa#1, dafa#2, and dafa#3 at 100 nM each, error bars, STD; ***P < 0.0001; **P < 0.001; *P < 0.05. B) Time point of 1st egg laying in daf-22(ok693) mutant worms treated with indicated concentrations of dafa#3; error bars, STD; ***P < 0.0001; **P < 0.001; *P < 0.05. For full data, see S14 Table. (TIF) [file pgen.1006717.s006.tif]

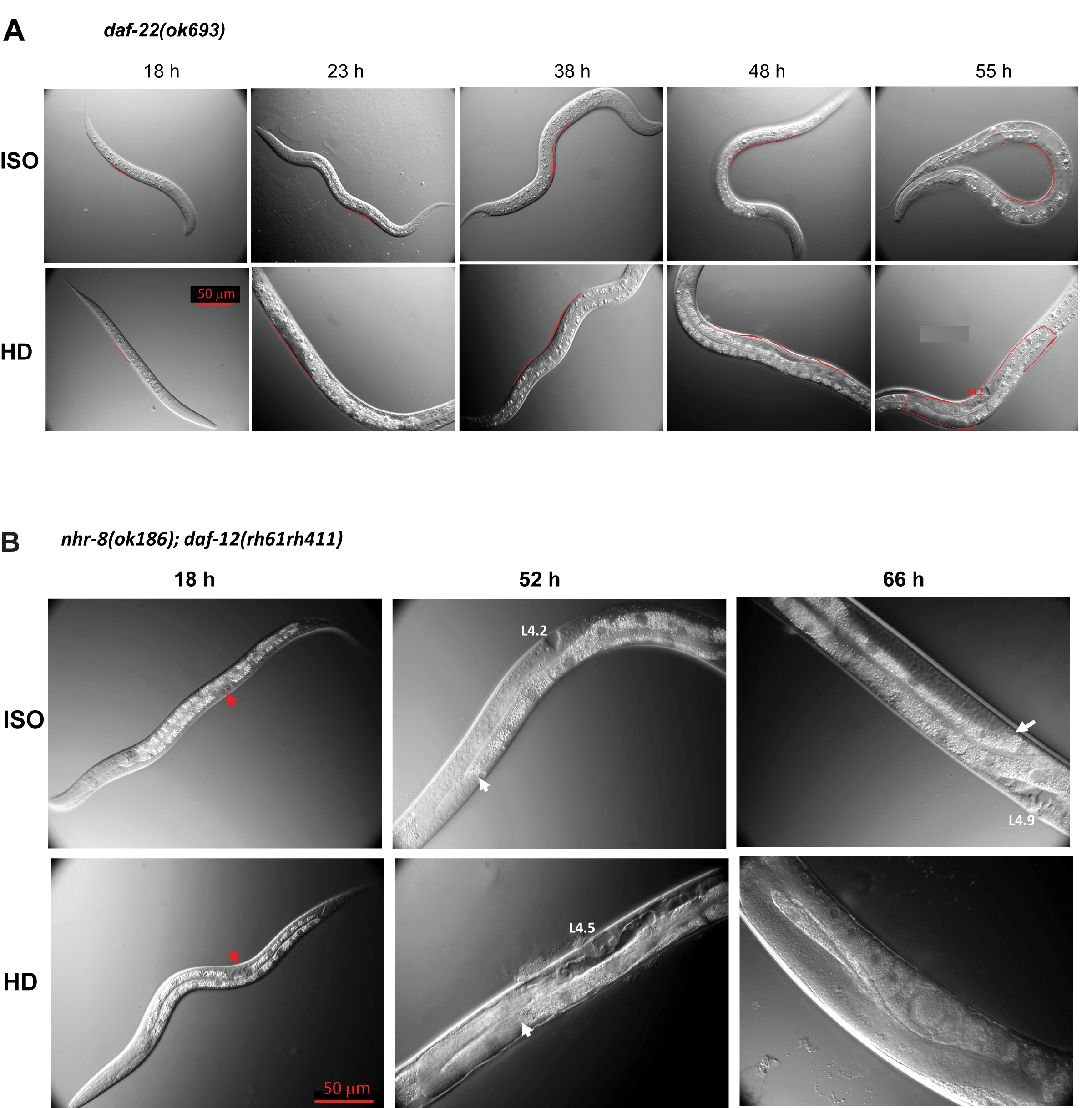

Supplement: S7 Fig — Images show daf-22(ok693) (A) and nhr-8(ok186);daf-12(rh61rh411) (B) mutant worms grown in isolation (ISO, upper panel) or in groups of >50 animals per plate (HD, lower panels). Red arrows and red lines indicate the position of the developing gonad, white arrows indicate the distal tip cell of the gonad (DTC), white numbers refer to sub-stages of larval stage 4, characterized by shape of the vulva and degree of gonadal reflection. (TIF) [file pgen.1006717.s007.tif]

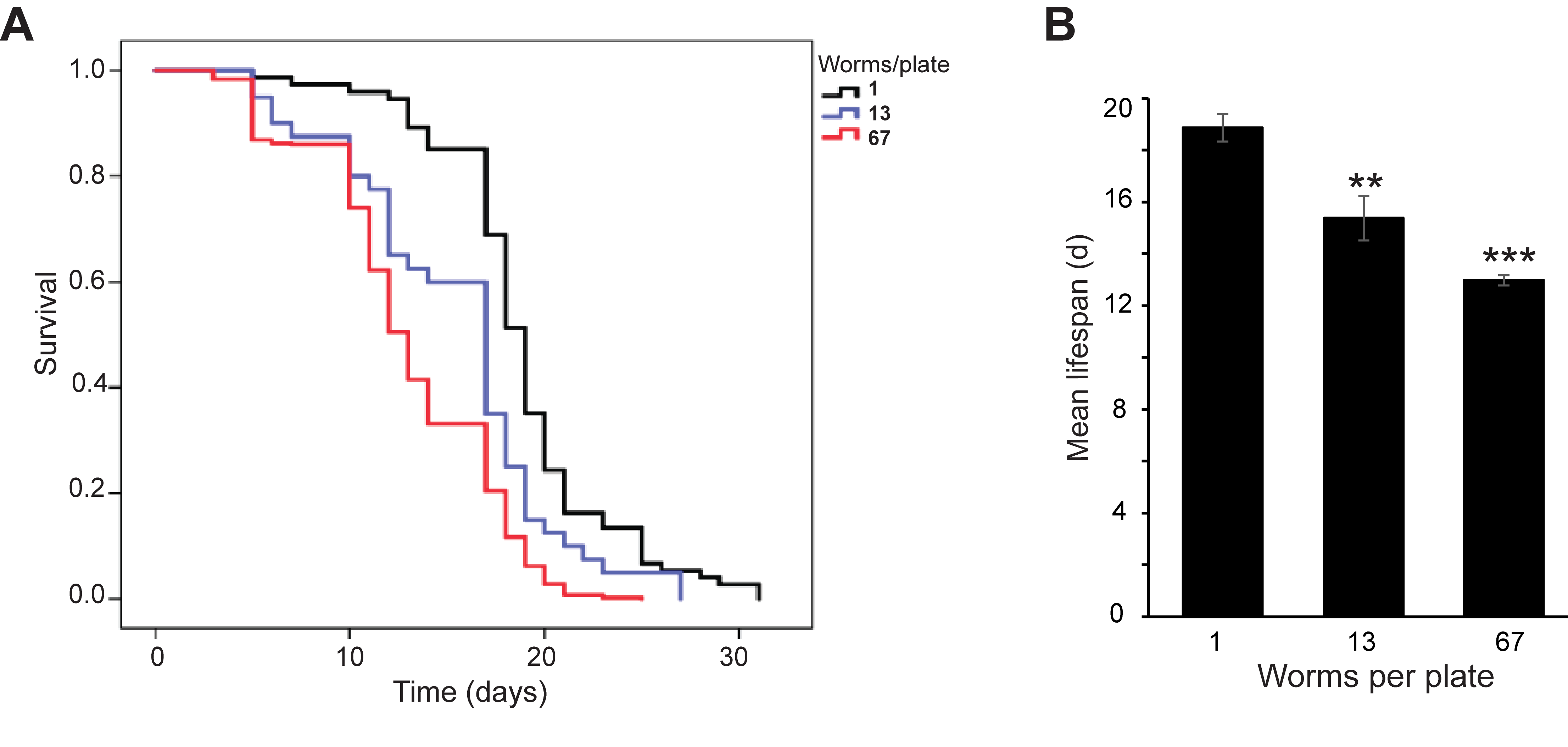

Supplement: S8 Fig — A) Survival curves (generated in SPSS) of 67, 13, and 1 worms/plate set up as eggs (Protocol B), without ethanol on the plates (ethanol was used for the lifespan data presented in Fig 4 and S9 Fig). B) Mean lifespan of worms in A. Error bars, SEM; ***P < 0.0001; **P < 0.001; *P < 0.05. For full data, see S16 Table. (TIF) [file pgen.1006717.s008.tif]

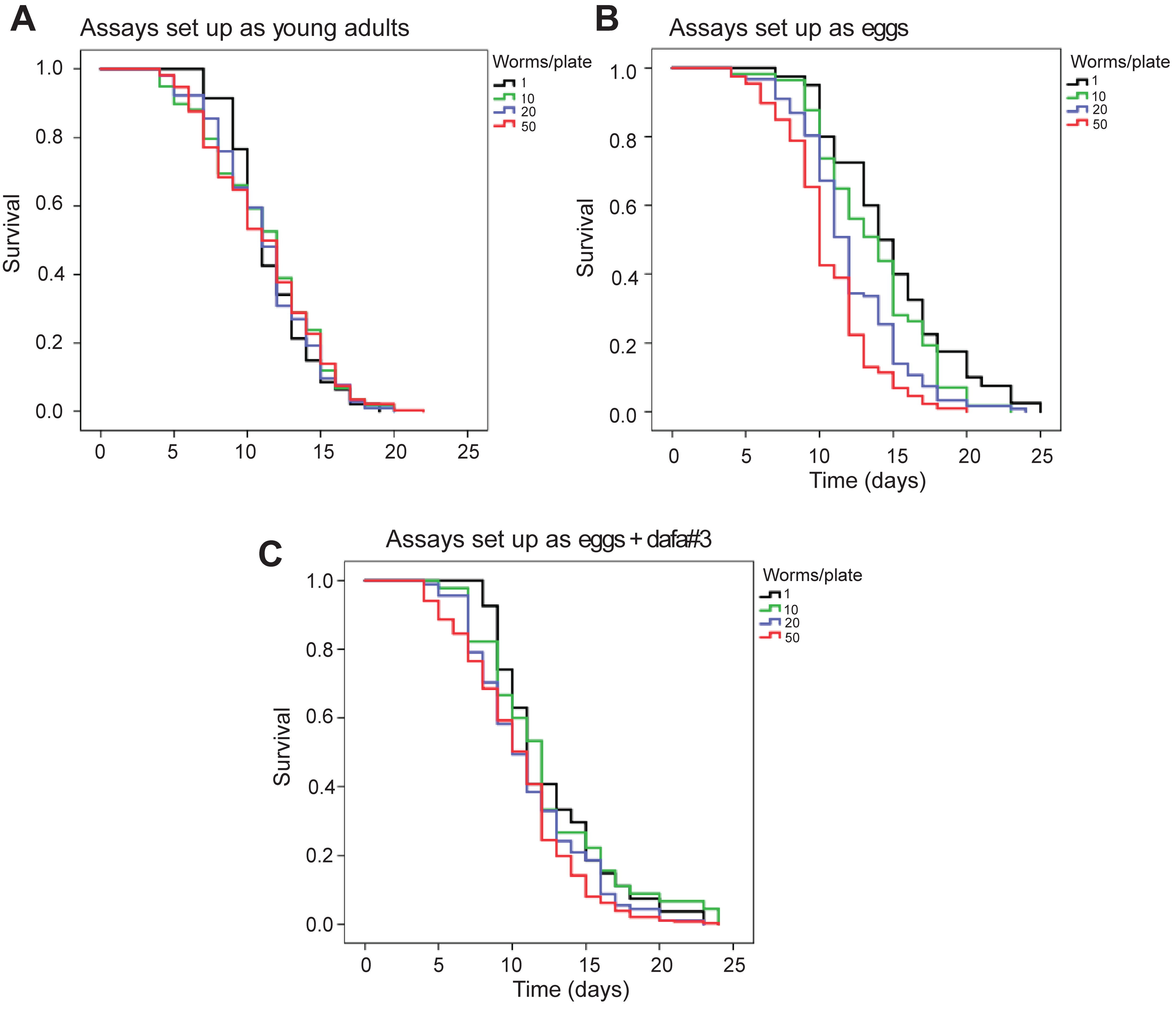

Supplement: S9 Fig — Survival curves (generated in SPSS, mean lifespans shown in Fig 4A) of A) worms set up as Young Adults at densities of 1, 10, 20, and 50 worms per plate (wpp); B) worms set up as eggs; C) worms set up as eggs using 100 nM dafa#3. Plates used for the experiments in A and B were mock-treated with the same amount of ethanol used in the preparation of dafa#3-containing plates in C. For full data, see S17 Table. (TIF) [file pgen.1006717.s009.tif]

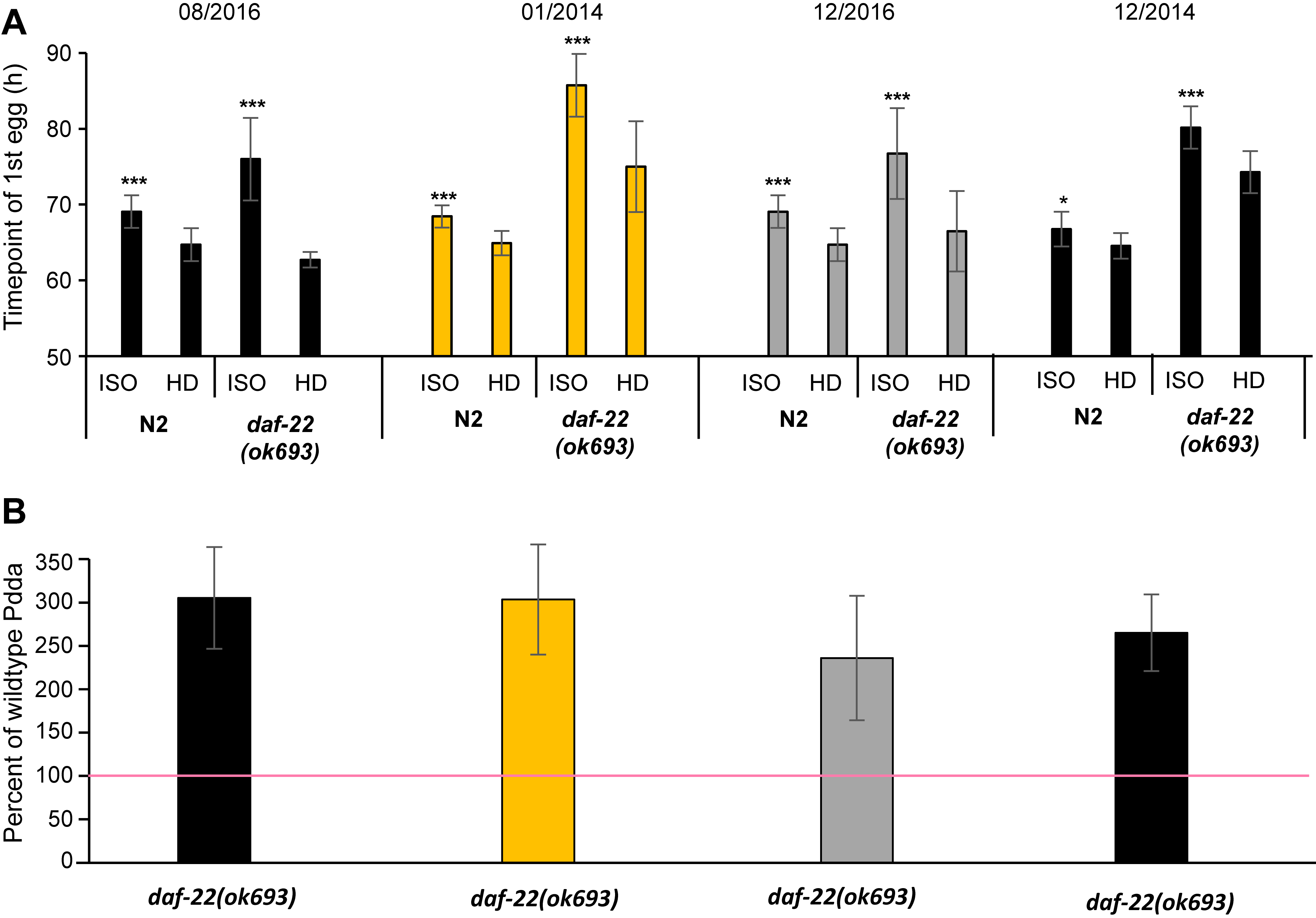

Supplement: S10 Fig — A) Timepoint of first egg laying (hours) in four separate experiments run at different times from 2014–2016; ISO: isolated as eggs; HD: grown at high density, isolated after 59 hours; B) Same data represented in terms of the Pdda metric; red line indicates 100% of wildtype Pdda. Error bars: STD, ***P < 0.0001; **P < 0.001; *P < 0.05. (TIF) [file pgen.1006717.s010.tif]
