# Supplementary material for: Larval crowding accelerates C. elegans development and reduces lifespan
Source: PLoS Genet. 2017 Apr 10;13(4):e1006717. doi: 10.1371/journal.pgen.1006717 (PMC5402976; doi:10.1371/journal.pgen.1006717)
Supplement: S1 Table — Comparison of protocols A and B (data shown in Fig 1A and 1C). ISO: isolation (1 worm per plate), HD; high density, wpp = worms per plate. a p-values for comparison of 10 wpp, 41 wpp, 242 wpp, 909 wpp with ISO (protocol A), and for comparison of HD61h and HD48h with ISO (protocol B). bplated with a worm sorter (COPAS Biosort, Union Biometra, Geel, Belgium). (DOCX) [file pgen.1006717.s011.docx]

| **Condition** | **Time of 1^st^ egg lay [h] (STD)** | **ΔISO-HD  [h] (STD)** | **%ISO (STD)** | **P value^a^** | **P value** |
| --- | --- | --- | --- | --- | --- |
| Protocol A, ISO | 73.91 (3.65) |  | 100 (4.9) |  |  |
| Protocol A, 10 wpp | 68.67 (2.09) | 5.2 (0.63) | 92.9 (12.1) | 9.38E-09 |  |
| Protocol A, 41 wpp | 65.08 (2.5) | 8.8 (0.77) | 88.05 (8.75) | 9.8E-10 | 10 wpp vs.  41 wpp  0.00044 |
| Protocol A, 242 wpp^b^ | 62.9 (1.7) | 11.01 (3.18) | 85.04 (4.3) | 7.9E-10 | 41 wpp vs.  242 wpp  0.016 |
| Protocol A, 909 wpp^b^ | 62.2 (1.17) | 11.7 (1.66) | 84.15 (1.58) | 2.7E-06 | 242 wpp vs.  909 wpp  0.51 |
|  |  |  |  |  |  |
| Protocol B, ISO | 71.7 (1.89) |  | 100 (2.28) |  |  |
| Protocol B, HD for 61 h | 65.0 (1.4) | 6.7 (0.74) | 90.7 (11.04) | 8.7E-08 |  |
| Protocol B, HD for 48 h | 67.23 (2.08) | 4.47 (0.83) | 93.8 (18.6) | 2.76E-05 | HD61h vs. HD48h  0.0035 |
