# Supplementary material for: Larval crowding accelerates C. elegans development and reduces lifespan
Source: PLoS Genet. 2017 Apr 10;13(4):e1006717. doi: 10.1371/journal.pgen.1006717 (PMC5402976; doi:10.1371/journal.pgen.1006717)
Supplement: S2 Table — Developing larvae were isolated at different time points during development, indicated as hours after synchronization. Data shown in S1 Fig, part F. a p-values for comparison of ISO, 8 h; ISO, 24 h; ISO, 39 h; ISO, 54 h and ISO, 61 h with ISO, 0 h (protocol B). (DOCX) [file pgen.1006717.s012.docx]

| **Condition** | **Time of 1^st^ egg lay [h] (STD)** | **ΔISO-HD**  **[h] (STD)** | **%ISO (STD)** | **P value^a^** |
| --- | --- | --- | --- | --- |
| ISO, 0 h | 67.08 (0.38) | - | 100 (4.8) |  |
| ISO, 8 h | 67.13 (0.13) | -0.05 (2.27) | 100.07 (3.7) | 0.96 |
| ISO, 24 h | 66.78 (0.55) | 0.3 (4.28) | 99.54 (4.05) | 0.729 |
| ISO, 39 h | 65.96 (0.26) | 1.12 (1.56) | 98.32 (4.1) | 0.2 |
| ISO, 54 h | 64.625 (0.45) | 2.46 (1.47) | 96.34 (3.1) | 0.0031 |
| ISO, 61 h | 64.2 (0.15) | 2.88 (2.2) | 95.71 (2.2) | 0.00033 |
