# Supplementary material for: Larval crowding accelerates C. elegans development and reduces lifespan
Source: PLoS Genet. 2017 Apr 10;13(4):e1006717. doi: 10.1371/journal.pgen.1006717 (PMC5402976; doi:10.1371/journal.pgen.1006717)
Supplement: S3 Table — (DOCX) [file pgen.1006717.s013.docx]

| **Developmental stage** | **ISO (%)** | **HD (%)** |
| --- | --- | --- |
| late L3 | 1.7 | 0 |
| moult L3/L4 | 5.2 | 1.6 |
| early L4 | 77.6 | 45.9 |
| mid L4 | 15.5 | 49.3 |
| late L4 | 0 | 1.6 |
| moult L4/ad | 0 | 1.6 |
