# Supplementary material for: Larval crowding accelerates C. elegans development and reduces lifespan
Source: PLoS Genet. 2017 Apr 10;13(4):e1006717. doi: 10.1371/journal.pgen.1006717 (PMC5402976; doi:10.1371/journal.pgen.1006717)
Supplement: S4 Table — Data are shown in Fig 1E and S3 Fig. ISO: isolation (1 worm per plate), HD; high density (50–100 worms per plate). bPdda assays with nhr-49(nr2041), hsf-1(sy441) and sir-2.1(ok434) mutants were performed only once, with >12 plates each for ISO and HD conditions. (DOCX) [file pgen.1006717.s014.docx]

| **Strain, condition** | **Time of 1^st^ egg lay [h] (STD)** | **Δ ISO-HD [h] (STD)** | **Time of first egg of HD worms as % of ISO worms (STD)** | **Percent of wildtype  Pdda (STD)** | **P-value**  **ISO/HD** | **P-value**  **N2/mutant** |
| --- | --- | --- | --- | --- | --- | --- |
| N2 ISO | 71.55 (2.68) |  |  |  |  |  |
| N2 HD | 67.41 (3.08) | 4.14 (0.52) | 94.21 (4.3) | 100 (12.6) | 2.0E-07 |  |
| *eat-2(ad465)* ISO | 76.23 (6.11) |  |  |  |  |  |
| *eat-2(ad465)* HD | 71.38 (6.03) | 4.85 (1.13) | 94.6 (7.9) | 109.96 (27.3) | 1.62E-06 | 0.595 |
|  |  |  |  |  |  |  |
| N2 ISO | 71.43 (2.4) |  |  |  |  |  |
| N2 HD | 67.58 (3.16) | 3.86 (0.7) | 94.61 (4.4) | 100(18.1) | 5.22E-05 |  |
| *egl-4(n477)* ISO | 68.13 (3.66) |  |  |  |  |  |
| *egl-4(n477)* HD | 64.43 (3.99) | 3.7 (1.2) | 94.6 (25.9) | 99.7 (31.08) | 0.0027 | 0.934 |
|  |  |  |  |  |  |  |
| N2 ISO^b^ | 72.75 (1.17) |  |  |  |  |  |
| N2 HD^b^ | 69.19 (2.45) | 3.56 (0.63) | 95.6 (3.7) | 100 (17.7) | 0.0022 |  |
| *nhr-49(nr2041)* ISO^b^ | 74.25 (1.75) |  |  |  |  |  |
| *nhr-49(nr2041)* HD^b^ | 70.4 (4.2) | 3.85 (1.47) | 94.81 (5.65) | 106 (41.3) | 0.023 | 0.883 |
|  |  |  |  |  |  |  |
| N2 ISO^b^ | 72.38 (2.0) |  |  |  |  |  |
| N2 HD^b^ | 68.7 (1.25) | 3.68 (0.81) | 94.9 (1.73) | 100 (22.01) | 0.0019 |  |
| *hsf-1(sy441)* ISO^b^ | 77.33 (2.0) |  |  |  |  |  |
| *hsf-1(sy441)* HD^b^ | 73.5 (5.5) | 3.83 (2.34) | 95.05 (7.13) | 97.4 (63.31) | 0.24 | 0.401 |
|  |  |  |  |  |  |  |
| N2 ISO^b^ | 67.36 (1.67) |  |  |  |  |  |
| N2 HD^b^ | 65.26 (1.667) | 2.1 (0.57) | 96.88 (2.5) | 100 (27.1) | 0.0018 |  |
| *sir-2.1(ok434)*ISO^b^ | 67.33 (3.62) |  |  |  |  |  |
| *sir-2.1(ok434)* HD^b^ | 64.83 (2.98) | 2.5 (1.17) | 96.29 (4.42) | 119 (55.7) | 0.056 | 0.428 |
