# Supplementary material for: Larval crowding accelerates C. elegans development and reduces lifespan
Source: PLoS Genet. 2017 Apr 10;13(4):e1006717. doi: 10.1371/journal.pgen.1006717 (PMC5402976; doi:10.1371/journal.pgen.1006717)
Supplement: S5 Table — Data shown in Fig 2A. ISO: isolation (1 worm per plate), HD; high density (50–100 worms per plate). (DOCX) [file pgen.1006717.s015.docx]

| **Strain, condition** | **Time of 1^st^ egg lay [h] (STD)** | **Δ ISO-HD [h] (STD)** | **Time of first egg of HD worms as % of ISO worms (STD)** | **Percent of wildtype  Pdda (STD)** | **P-value ISO/HD** | **P-value N2/mutant** |
| --- | --- | --- | --- | --- | --- | --- |
| N2 ISO | 68.71 (1.49) |  |  |  |  |  |
| N2 HD | 64.8 (1.7) | 3.9 (0.29) | 94.3 (2.44) | 100 (7.4) | 1.7E-25 |  |
| *daf-22(ok693)* ISO | 82.68 (5.9) |  |  |  |  |  |
| *daf-22(ok693)* HD | 70.42 (6.78) | 12.3 (1.1) | 85.2 (8.2) | 262.1 (28.2) | 1.01E-18 | 6.61E-23 |
| N2 ISO | 71.88 (3.34) |  |  |  |  |  |
| N2 HD | 69.67 (3.6) | 2.2 (0.77) | 96.9 (5.0) | 100 (35) | 0.0032 |  |
| *daf-22(m130)* ISO | 75.6 (5.4) |  |  |  |  |  |
| *daf-22(m130)* HD | 69.84 (4.5) | 5.76 (1.04) | 92.38 (5.9) | 248.9 (47) | 5.25E-06 | 0.0001 |
|  |  |  |  |  |  |  |
| N2 ISO | 71.8 (2.12) |  |  |  |  |  |
| N2 HD | 67.0 (2.42) | 4.8(+-0.57) | 93.3 (3.4) | 100 (11.9) | 8.17E-12 |  |
| *dhs-28(hj8)* ISO | 88.12 (7) |  |  |  |  |  |
| *dhs-28(hj8)* HD | 71.18 (4.7) | 16.94 (1.5) | 80.8 (5.3) | 287.6 (31.3) | 4.6E-17 | 2E-15 |
| N2 ISO | 71.99(2.1) |  |  |  |  |  |
| N2 HD | 67.16 (2.5) | 4.83 (0.5) | 93.3 (3.5) | 100 (10.4) | 1.4E-15 |  |
| *dhs-28(tm2581)* ISO | 95.25 (8.3) |  |  |  |  |  |
| *dhs-28(tm2581)* HD | 84.52 (4.6) | 10.7(1.5) | 88.7 (4.8) | 167.4 (31) | 1.9E-10 | 2E-06 |
