# Supplementary material for: Larval crowding accelerates C. elegans development and reduces lifespan
Source: PLoS Genet. 2017 Apr 10;13(4):e1006717. doi: 10.1371/journal.pgen.1006717 (PMC5402976; doi:10.1371/journal.pgen.1006717)
Supplement: S6 Table — Timepoint of 1st egg in N2 wildtype, daf-22(ok693) and in nhr-8(ok186) daf-12(rh61411) mutants grown on regular plates or on plates pre-populated with daf-22(ok693) or N2 wildtype larvae (data shown in Figs 2G, 3H, and S5 Fig). ISO: isolation (1 worm per plate), HD; high density (50–100 worms per plate); HDpreISO: assay with one worm per plate, using HD-pre-conditioned plates. Assays with daf-22(ok693): two independent biological repeats with >20 plates per condition; assays with N2 and nhr-8(ok186); daf-12(rh61rh411): one assay each with >20 plates per condition. (DOCX) [file pgen.1006717.s016.docx]

| **Strain, condition** | **Time of 1^st^ egg lay [h] (STD)** | **Δ ISO-HD [h] (STD)** | **Time of first egg of HD worms as % of ISO worms (STD)** | **P-value  ISO/HD or ISO/HDpreISO** | **P-value HD/HDpreISO** |
| --- | --- | --- | --- | --- | --- |
| Pre-incubation assay with *daf-22(ok693)* mutants using  *daf-22(ok693)-*preincubated plates |  |  |  |  |  |
| *daf-22(ok693)* ISO | 73.78 (4.65) |  |  |  |  |
| *daf-22(ok693)* HD | 67.46 (1.43) | 6.32 (5.1) | 91.3 (1.9) | 1.982E-13 |  |
| *daf-22(ok693)* HDpreISO | 70.59 (2.09) | 3.2 (4.7) | 95.7 (2.8) | 0.000235 | 2.28E-06 |
|  | | | | | |
| Pre-incubation assay with wildtype (N2) using  wildtype*-*preincubated plates |  |  |  |  |  |
| N2 ISO | 66.27 (1.4) |  |  |  |  |
| N2 HD | 64.05 (1.95) | 2.2 (2.2) | 96.7 (2.2) | 0.001216 |  |
| N2 HDpreISO | 64.0 (3.2) | 2.27 (2.4) | 96.6 (4.8) | 0.015 | 0.949 |
|  | | | | | |
| Pre-incubation assay with *nhr-8(ok186); daf-12 (rh61rh411)* mutants using *daf-22(ok693)-*preincubated plates |  |  |  |  |  |
| N2 ISO | 66.44 (3.01) |  |  |  |  |
| N2 HD | 64.76 (2.55) | 1.68 (2.6) | 100 (4.1) | 0.042 |  |
|  |  |  |  |  |  |
| *nhr-8(ok186); daf-12 (rh61rh411)* ISO | 72.11 (5.3) |  |  |  |  |
| *nhr-8(ok186); daf-12 (rh61rh411)* HD | 66.53 (3.2) | 5.58 (5.7) | 92.26 (4.8) | 0.000459 |  |
| *nhr-8(ok186); daf-12(rh61rh411*) HDpre (*daf-22(ok693*))ISO | 66.64 (3.0) | 5.47(+-5.1) | 92.41 (3.7) | 0.0002 | 0.399 |
| *pdda* for *nhr-8(ok186); daf-12 (rh61rh411)* mutant worms in this experiment: 306.02% (STD: 86.25%) | | | | | |
