# Supplementary material for: Larval crowding accelerates C. elegans development and reduces lifespan
Source: PLoS Genet. 2017 Apr 10;13(4):e1006717. doi: 10.1371/journal.pgen.1006717 (PMC5402976; doi:10.1371/journal.pgen.1006717)
Supplement: S7 Table — Data are shown in Fig 2D and 2E. ISO: isolation (1 worm per plate), HD; high density (50–100 worms per plate). aAssays with daf-22(ok693) and ascr#3: two independent biological repeats, each using >12 plates per assay, assays with daf-22(ok693) ascr#2: one data set, using >12 plates per condition. eEthanol containing plates (0.002% v/v) (DOCX) [file pgen.1006717.s017.docx]

| **Strain, condition** | **Time of 1^st^ egg lay [h] (STD)** | **Δ ISO-HD [h] (STD)** | **Time of first egg of HD worms as % of ISO**  **worms (STD)** | **Percent of Pdda in mock treated worms (STD)** | **P-value ISO/HD** | **P-value control vs. treatment** |
| --- | --- | --- | --- | --- | --- | --- |
| N2 ISO^e^ | 70.76 (1.4) |  |  |  |  |  |
| N2 HD^e^ | 66.92 (1.34) | 3.84 (0.4) | 94.57 (2.64) | 100 (10.4) | 9.5E-08 |  |
| N2 ascr#2 ISO ^e^ | 68.95 (1.5) |  |  |  |  |  |
| N2 ascr#2 HD ^e^ | 68.08 (2.4) | 0.87 (0.59) | 98.74 (3.51) | 23.3 (15.4) | 0.162 | 0.0016 |
| N2 ISO^e^ | 68.94 (3.2) |  |  |  |  |  |
| N2 HD^e^ | 65.49 (2.25) | 3.45 (0.55) | 95.0 (3.3) | 100 (15.9) | 2.38E-09 |  |
| N2 ascr#3 ISO^e^ | 67.7 (2.4) |  |  |  |  |  |
| N2 ascr#3 HD ^e^ | 65.77 (2.33) | 1.9 (0.48) | 97.14 (3.44) | 56.1 (13.9) | 0.0109 | 0.00063 |
|  |  |  |  |  |  |  |
| *daf-22(ok693)* ISO^e^ | 85.76 (3.07) |  |  |  |  |  |
| *daf-22(ok693)* HD^e^ | 78.71 (2.91) | 7.05 (0.66) | 91.78 (3.4) | 100 (9.36) | 1.7 E-12 |  |
| *daf-22(ok693*) ascr#2 ISO^a e^ | 81.37 (4.0) |  |  |  |  |  |
| *dhs-22(ok693)* ascr#2 HD^a,e^ | 80.5 (3.6) | 0.87 (1.3) | 98.93 (4.5) | 12.3 (18.4) | 0.51 | 5.6 E-06 |
| *daf-22(ok693)* ascr#3 ISO^a,e^ | 83.89 (3.04) |  |  |  |  |  |
| *daf-22(ok693)* ascr#3 HD^a,e^ | 83.48 (3.47) | 0.4 (0.8) | 99.5 (4.12) | 5.7 (11.3) | 0.69 | 1.144E-07 |
