# Supplementary material for: Larval crowding accelerates C. elegans development and reduces lifespan
Source: PLoS Genet. 2017 Apr 10;13(4):e1006717. doi: 10.1371/journal.pgen.1006717 (PMC5402976; doi:10.1371/journal.pgen.1006717)
Supplement: S8 Table — Data are shown in Fig 2F. ISO: isolation (1 worm per plate), HD; high density (50–100 worms per plate). (DOCX) [file pgen.1006717.s018.docx]

| **Strain, condition** | **Time of 1^st^ egg lay [h] (STD)** | **Δ ISO-HD [h] (STD)** | **Time of first egg of HD worms as % of  ISO worms (STD)** | **Percent of wildtype  Pdda (STD)** | **P-value ISO/HD** | **P-value N2/mutant** |
| --- | --- | --- | --- | --- | --- | --- |
| N2 ISO | 70.0 ( 2.7) |  |  |  |  |  |
| N2 HD | 67.23 (0.9) | 2.7 (0.39) | 96.04 (3.4) | 100 (14.4) | 6.53E-08 |  |
| *odr-3(n2150)* ISO | 70.53 (3.3) |  |  |  |  |  |
| *odr-3(n2150)* HD | 67.74 (2.9) | 2.79(0.62) | 96.04 (4.1) | 103 (23) | 1.25E-06 | 0.777 |
|  |  |  |  |  |  |  |
| N2 ISO | 69.07 (2.4) |  |  |  |  |  |
| N2 HD | 66.52 (1.7) | 2.55 (0.43) | 96.3 (2.5) | 100 (16.9) | 1.13E-07 |  |
| *tax-4(ks28)* ISO | 78.49 ( 5.6) |  |  |  |  |  |
| *tax-4(ks28)* HD | 68.48 (4.5) | 10.01 (1.0) | 87.25 (5.8) | 345.4 (39.2) | 2.2E-15 | 1.38E-15 |
| *osm-6(p811)* ISO | 75.6 (6.2) |  |  |  |  |  |
| *osm-6(p811)* HD | 66.72 (2.8) | 8.8 (0.96) | 88.25 (3.8) | 315.3 (37.6) | 3.4E-13 | 4.69E-20 |
|  |  |  |  |  |  |  |
| N2 ISO | 72.05 ( 3.9) |  |  |  |  |  |
| N2 HD | 68.53 (5.2) | 3.52 (1.6) | 95.1 (1.6) | 100 (45.5) | 0.04 |  |
| *mec-4(e1339)* ISO | 69.47 ( 4.373) |  |  |  |  |  |
| *mec-4(e1339)* HD | 66.81 (3.792) | 2.66 (0.99) | 96.17 (1.4) | 78.4 (28.13) | 0.017 | 0.239 |
