# Supplementary material for: Larval crowding accelerates C. elegans development and reduces lifespan
Source: PLoS Genet. 2017 Apr 10;13(4):e1006717. doi: 10.1371/journal.pgen.1006717 (PMC5402976; doi:10.1371/journal.pgen.1006717)
Supplement: S9 Table — Assays were run using protocol B using plates containing 400 nM ascr#2, ascr#3, ascr#10 or mock treatment (data shown in S4 Fig). ISO: isolation (1 worm per plate), HD; high density (50–100 worms per plate). eEthanol containing plates (0.002% v/v). (DOCX) [file pgen.1006717.s019.docx]

| **Strain, condition** | **Time of 1^st^ egg lay [h] (STD)** | **Δ ISO-HD [h] (STD)** | **Time of first egg of HD worms as % of ISO worms (STD)** | **Percent of wildtype  Pdda (STD)** | **P-value ISO/HD** | **P-value N2 vs. mutant** | **P-value control vs. treatment** |
| --- | --- | --- | --- | --- | --- | --- | --- |
| N2 ISO^e^ | 67.99 (2.78) |  |  |  |  |  |  |
| N2 HD^e^ | 64.95 (1.48) | 3.04 (0.68) | 95.46 (2.05) | 100 (22.4) | 0.00014 |  |  |
| N2 ascr#2 ISO ^e^ | 67.754 (1.58) |  |  |  |  |  |  |
| N2 ascr#2 HD^e^ | 67.952 (2.87) | -0.198 (0.72) | 100.29 (5.02) | -6.53 (23.7) | 0.834 |  | 0.00064 |
| N2 ascr#3 ISO^e^ | 66.68 (2.98) |  |  |  |  |  |  |
| N2 ascr#3 HD^e^ | 64.81 (2.72) | \| 1.87 (0.9) \| \| --- \| \|  \| | 97.26 (4.1) | 64.53 (28.62) | 0.039 |  | 0.11 |
| N2 ascr#10, ISO^e^ | 68.71 (2.43) |  |  |  |  |  |  |
| N2 ascr#1 HD^e^ | 66.24 (2.84) | 2.47 (0.82) | 96.4 (4.14) | 80.4 (26.97) | 0.0043 |  | 0.496 |
|  |  |  |  |  |  |  |  |
| *tax-4(ks28)* ISO^e^ | 73.63 (3.2) |  |  |  |  |  |  |
| *tax-4(ks28)* HD^e^ | 66.25 (0.97) | 7.38 (0.68) | 89.98 (1.4) | 224.2 (22.4) | 5.76E-11 | 5.29E-06 |  |
| *tax-4(ks28)* ascr#2 ISO^e^ | 73.48 (1.47) |  |  |  |  |  |  |
| *tax-4(ks28)* ascr#2 HD^e^ | 66.46 (1.02) | 7.02 (0.37) | 90.45 (1.6) | 213.7 (12.2) | 4.2E-22 | 4.6E-07 | 0.872 |
| *osm-6(p811)* ISO^e^ | 70.825 (2.8) |  |  |  |  |  |  |
| *osm-6(p811)* HD^e^ | 64.875 (1.4) | 5.95 (0.65) | 91.6 (1.97) | 187.9 (21.4) | 0.09E-11 | 0.00082 |  |
| *osm-6(p811) ascr#2* ISO^e^ | 70.54 (1.82) |  |  |  |  |  |  |
| *osm-6(p811) ascr#2* HD^e^ | 64.35 (1.09) | 6.19 (0.44) | 91.22 (1.54) | 196.3 (14.5) | 8.24E-17 | 4.11E-05 | 0.739 |
