# Supplementary material for: Larval crowding accelerates C. elegans development and reduces lifespan
Source: PLoS Genet. 2017 Apr 10;13(4):e1006717. doi: 10.1371/journal.pgen.1006717 (PMC5402976; doi:10.1371/journal.pgen.1006717)
Supplement: S10 Table — Assays were run using protocol B (data shown in Fig 3B). ISO: isolation (1 worm per plate), HD; high density (50–100 worms per plate). (DOCX) [file pgen.1006717.s020.docx]

| **Strain, condition** | **Time of 1^st^ egg lay [h] (STD)** | **Δ ISO-HD [h] (STD)** | **Time of first egg of HD worms as % of ISO worms (STD)** | **Percent of wildtype  Pdda (STD)** | **P-value ISO/HD** | **P-value N2/mutant** |
| --- | --- | --- | --- | --- | --- | --- |
| N2 ISO | 70.58 (2.4) |  |  |  |  |  |
| N2 HD | 66.64 (1.8) | 3.9 (0.42) | 94.4 (2.55) | 100 (10.8) | 1.7E-16 |  |
| *daf-2(e1368)* ISO | 69.93 (3.1) |  |  |  |  |  |
| *daf-2(e1368)* HD | 70.12 (2.8) | -0.19 (0.58) | 100.3 (3.95) | -4.6 (14.9) | 0.68 | 5.63E-06 |
|  |  |  |  |  |  |  |
| N2 ISO | 70.74 (1.44) |  |  |  |  |  |
| N2 HD | 66.92 (1.74 | 3.82 (0.5) | 94.6 (1.99) | 100 (13.1) | 9.53E-08 |  |
| *ins-11(tm1053)* ISO | 69.4 (1.3) |  |  |  |  |  |
| *ins-11(tm1053)* HD | 65.9 (1.8) | 3.5 (0.52) | 94.96 (2.7) | 93.4 (13.6) | 1.77E-06 | 0.61 |
|  |  |  |  |  |  |  |
| N2 ISO | 72.87(2.63) |  |  |  |  |  |
| N2 HD | 68.92 (3.2) | 3.95 (0.66) | 94.5 (4.4) | 100 (16.7) | 1.4E-07 |  |
| *daf-16(mu86)* ISO | 69.7 (3.7) |  |  |  |  |  |
| *daf-16(mu86)* HD | 70.49 (2.8) | -0.79 (0.75) | 101.13 (4.0) | -20.9 (19) | 0.296 | 5.0E-09 |
|  |  |  |  |  |  |  |
| N2 ISO | 72.76 (2.6) |  |  |  |  |  |
| N2 HD | 69.18 (2.8) | 3.58 (0.44) | 95.08 (3.9) | 100 (12.3) | 2.1E-08 |  |
| *daf-16(m26)* ISO | 68.84 (2.7) |  |  |  |  |  |
| *daf-16(m26)* HD | 68.4 (3.3) | 0.44 (0.56) | 99.36 (4.73) | 12.99 (15.6) | 0.512 | 2.733E-05 |
