# Supplementary material for: Larval crowding accelerates C. elegans development and reduces lifespan
Source: PLoS Genet. 2017 Apr 10;13(4):e1006717. doi: 10.1371/journal.pgen.1006717 (PMC5402976; doi:10.1371/journal.pgen.1006717)
Supplement: S11 Table — ISO: isolation (1 worm per plate), HD; high density (50–100 worms per plate). (DOCX) [file pgen.1006717.s021.docx]

| **Strain, condition** | **Time of 1^st^ egg lay [h] (STD)** | **Δ ISO-HD [h] (STD)** | **Time of first egg of HD worms as % of ISO worms (STD)** | **Percent of wildtype  Pdda (STD)** | **P-value ISO/HD** | **P-value N2/mutant** |
| --- | --- | --- | --- | --- | --- | --- |
| N2 ISO | 68.84 (2.29) |  |  |  |  |  |
| N2 HD | 66.04 (2.01) | 2.8 (0.5) | 95.93 (2.9) | 100 (17.9) | 8.13E-08 |  |
| *daf-2(e1370)* ISO | 98.43 (7.27) |  |  |  |  |  |
| *daf-2(e1370)* HD | 98.8 (6.92) | -0.37(1.56) | 100.38 (7.03) | -9.24 (55.71) | 0.81 | 0.0016 |
|  |  |  |  |  |  |  |
| N2 ISO | 68.83 (1.85) |  |  |  |  |  |
| N2 HD | 65.92 (1.5) | 2.91 (0.68) | 95.77 (2.2) | 100 (23.36) | 0.00015 |  |
| *daf-2(e1368)* ISO | 70.33 (1.27) |  |  |  |  |  |
| *daf-2(e1368)* HD | 70.33 (1.56) | 0 (0.44) | 100 (2.22) | 0 (15.12) | 1 | 0.00014 |
|  |  |  |  |  |  |  |
| N2 ISO | 69.69 (2.72) |  |  |  |  |  |
| N2 HD | 65.82 (2.11) | 3.87 (0.83) | 94.44 (1.93) | 100 (21.44) | 0.00023 |  |
| *daf-2(m577)* ISO | 67.73 (2.22) |  |  |  |  |  |
| *daf-2(m577)* HD | 68.27 (2.6) | -0.54 (0.71) | 100.79 (3.84) | -14.36 (18.3) | 0.55 | 5.88E-05 |
