# Supplementary material for: Larval crowding accelerates C. elegans development and reduces lifespan
Source: PLoS Genet. 2017 Apr 10;13(4):e1006717. doi: 10.1371/journal.pgen.1006717 (PMC5402976; doi:10.1371/journal.pgen.1006717)
Supplement: S12 Table — Assays were run using protocol B (data shown in Fig 3C). ISO: isolation (1 worm per plate), HD; high density (50–100 worms per plate). (DOCX) [file pgen.1006717.s022.docx]

| **Strain, condition** | **Time of 1^st^ egg lay [h] (STD)** | **Δ ISO-HD [h] (STD)** | **Time of first egg of HD worms as % of ISO worms (STD)** | | **Percent of wildtype  Pdda (STD)** | **P-value ISO/HD** | **P-value N2/mutant** |
| --- | --- | --- | --- | --- | --- | --- | --- |
| N2 ISO | 68.51 (1.8) |  |  | |  |  |  |
| N2 HD | 65.84 (2.4) | 2.67 (0.35) | 96.1 (3.4) | | 100 (13.1) | 5.8E-13 |  |
| *daf-12(rh61rh411)* ISO | 66.81 (3.3) |  |  | |  |  |  |
| *daf-12(rh61rh411)* HD | 65.33 (3.4) | 1.48 (0.53) | 97.78 (5.09) | | 56.8 (19.9) | 0.000794 | 0.0153 |
|  |  |  |  | |  |  |  |
| N2 ISO | 68.16 (2.7) |  |  | |  |  |  |
| N2 HD | 64.48 (2.1) | 3.68 (0.44) | 94.61 (3.07) | | 100 (11.99) | 3.08E-13 |  |
| *daf-9(rh50)* ISO | 69.68 (3.52) |  |  | |  |  |  |
| *daf-9(rh50)* HD | 67.97 (5.66) | 1.71 (0.9) | 97.55 (8.1) | | 45.45 (24.5) | 0.087 | 0.002 |
|  |  |  |  | |  |  |  |
| N2 ISO | 67.53 (3.65) |  |  | |  |  |  |
| N2 HD | 64.32 (2.8) | 3.2 (0.36) | 95.25 (4.07) | | 100 (11.3) | 3.74E-17 |  |
| *daf-12(rh61rh411) daf-9(rh50)* ISO | 72.11 (3.7) |  |  | |  |  |  |
| *daf-12(rh61rh411)* *daf-9(rh50)* HD | 69.43 (4.1) | 2.68 (0.46) | 96.28 (5.6) | | 78.43 (+- 14.4) | 1.59E-08 | 0.19 |
|  |  |  |  | |  |  |  |
| N2 ISO | 71.99 (2.1) |  | |  |  |  |  |
| N2 HD | 67.16 (2.5) | 4.83 (0.47) | | 93.29 (3.5) | 100 (9.7) | 2.3E-13 |  |
| *nhr-8(tm1800)* ISO | 72.31 (4.5) |  | |  |  |  |  |
| *nhr-8(tm1800)* HD | 71.7 (6.1) | 0.61 (1.08) | | 99.16 (8.4) | 12.6 (22.4) | 0.597 | 2.5E-05 |
|  |  |  | |  |  |  |  |
| N2 ISO | 70.06 (5.5) |  | |  |  |  |  |
| N2 HD | 66.98 (5.0) | 3.08(0.61) | | 95.6 (3.8) | 100 (19.8) | 1.04E-06 |  |
| *nhr-8(ok186)* ISO | 67.32 (3.2) |  | |  |  |  |  |
| *nhr-8(ok186)* HD | 66.9 (3.1) | 0.42 (0.46) | | 99.38 (4.67) | 14.2 (13.6) | 0.4 | 1.43E-06 |
