# Supplementary material for: Larval crowding accelerates C. elegans development and reduces lifespan
Source: PLoS Genet. 2017 Apr 10;13(4):e1006717. doi: 10.1371/journal.pgen.1006717 (PMC5402976; doi:10.1371/journal.pgen.1006717)
Supplement: S13 Table — Assays were run using protocol B. ISO: isolation (1 worm per plate), HD; high density (50–100 worms per plate). *same control as in S11 Table* (daf-2(e1370)); **same control as in S11 Table* (daf-2(e1368)) (DOCX) [file pgen.1006717.s023.docx]

| **Strain, condition** | **Time of 1^st^ egg lay [h] (STD)** | **Δ ISO-HD [h] (STD)** | **Time of first egg of HD worms as % of ISO worms (STD)** | **Percent of wildtype  Pdda (STD)** | **P-value ISO/HD** | **P-value N2/mutant** |
| --- | --- | --- | --- | --- | --- | --- |
| N2 ISO | 69.125 (2.83) |  |  |  |  |  |
| N2 HD | 65.462(2.62) | 3.66 (0.84) | 94.7 (4.24) | 100 (23.0) | 0.0022 |  |
| *nhr-8(ok186)* ISO | 66.18 (2.95) |  |  |  |  |  |
| *nhr-8(ok186)* HD | 66.13 (2.48) | 0.05 (0.62) | 99.93 (3.74) | 1.43 (16.94) | 0.52 | 0.00039 |
|  |  |  |  |  |  |  |
| N2 ISO* | 68.84 (2.29) |  |  |  |  |  |
| N2 HD* | 66.04 (2.01) | 2.8 (0.5) | 95.93 (2.9) | 100 (17.9) | 8.13E-08 |  |
| *nhr-8(tm1800)* ISO | 70.39 (2.35) |  |  |  |  |  |
| *nhr-8(tm1800)* HD | 70.16 (1.8) | 0.23 (0.5) | 99.66 (2.55) | 8.03 (17.86) | 0.68 | 7.1E-05 |
|  |  |  |  |  |  |  |
| N2 ISO** | 68.83 (1.85) |  |  |  |  |  |
| N2 HD** | 65.92 (1.5) | 2.91 (0.68) | 95.77 (2.2) | 100 (23.36) | 0.00015 |  |
| *nhr-8(hd117)* ISO | 67.4 (1.74) |  |  |  |  |  |
| *nhr-8(hd117)* HD | 67.0 (1.51) | 0.4 (0.42) | 99.4 (2.2) | 14.04 (14.4) | 0.354 | 0.0009 |
