# Supplementary material for: Larval crowding accelerates C. elegans development and reduces lifespan
Source: PLoS Genet. 2017 Apr 10;13(4):e1006717. doi: 10.1371/journal.pgen.1006717 (PMC5402976; doi:10.1371/journal.pgen.1006717)
Supplement: S14 Table — Worms were mock treated or grown in presence of dafachronic acids at 100 nM (or indicated concentrations), using protocol B. ISO: isolation (1 worm per plate), HD; high density (50–100 worms per plate). Data shown in S6 Fig. aAssays with daf-22(ok693) and dafa#1 and dafa#2: one data set each with >12 plates per condition. ePlates with EtOH (0.2% v/v). (DOCX) [file pgen.1006717.s024.docx]

| **Strain, condition** | **Time of 1^st^ egg lay [h] (STD)** | **Δ ISO-HD [h] (STD)** | **Time of first egg of HD worms as % of ISO worms (STD)** | **Percent of wildtype  Pdda (STD)** | **P-value ISO/HD** | **P-value  control/ treatment** |
| --- | --- | --- | --- | --- | --- | --- |
| N2 ISO^e^ | 66.01 (2.04) |  |  |  |  |  |
| N2 HD^e^ | 63.56 (1.8) | 2.45 (0.24) | 96.3 (0.25) | 100 (9.8) | 5.1E-20 |  |
| N2 dafa1# ISO^e^ | 65.22(1.9) |  |  |  |  |  |
| N2, dafa# 1 HD^e^ | 65.57(2.4) | -0.35 (0.61) | 100.53 (3.6) | -14.5(24.9) | 0.57 | 1.83E-08 |
| N2, dafa#3 ISO ^e^ | 64.67 (2.5) |  |  |  |  |  |
| N2 HD dafa#3 HD^e^ | 64.37 (2.4) | 0.3 (0.48) | 99.5 (3.4) | 12.5 (19.6) | 0.14 | 1.092E-11 |
|  |  |  |  |  |  |  |
| *daf-22(ok693)* ISO^e^ | 81.75 (3.0) |  |  |  |  |  |
| *daf-22(ok693)* HD^e^ | 77.23 (1.1) | 4.52 (0.61) | 94.47 (1.3) | 100 (13.5) | 1.6E-07 |  |
| *daf-22(ok693)* dafa#1 ISO^a,e^ | 79.33(2.3) |  |  |  |  |  |
| *daf-22(ok693)* dafa#1 HD^a,e^ | 76.87(1.4) | 2.46 (0.69) | 96.89 (2.76) | 56.1 (15.3) | 0.0084 | 0.0013 |
| *daf-22(ok693)* dafa#2 ISO^a,e^ | 80 (2.0) |  |  |  |  |  |
| *daf-22(ok693)* dafa#2 HD^a,e^ | 79.86 (2.2) | 0.14(0.78) | 99.8 (2.77) | 3.1 (17.3) | 0.86 | 2.94E-06 |
| *daf-22(ok693)* dafa#3 ISO^e^ | 80.18 (2.5) |  |  |  |  |  |
| *daf-22(ok693*) dafa#3 HD^e^ | 79.29 (2.6) | 0.89(1.03) | 98.88 (3.2) | 20.1 (22.8) | 0.424 | 0.000217 |
| *daf-22(ok693)* DA Mix ISO^e^ | 78.75 (2.9) |  |  |  |  |  |
| *daf-22(ok693)* DA Mix HD ^e^ | 77.87 (2.8) | 0.88(0.68) | 98.9 (4.3) | 20.2 (15) | 0.23 | 8.92E-07 |
|  |  |  |  |  |  |  |
| *daf-22(ok693)* ISO^e^ | 83 (4.3) |  |  |  |  |  |
| *daf-22(ok693)* HD^e^ | 71.95 (2.4) | 11.05 (1.1) | 86.7 (2.89) | 100 (9.86) | 4.2E-11 |  |
| *daf-22(ok693)* dafa#3, 100 nM ISO^e^ | 72.5 (2.5) | 0.55 (1.06) | 87.3 (3.05) | 5.7 (9.6) | 0.586 | 5.9E-10 |
| *daf-22(ok693)* dafa#3, 10 nM ISO^e^ | 73.35 (2.9) | 1.4 (1.17) | 88.4 (3.5) | 14.3 (10.6) | 0.176 | 6.4E-07 |
| *daf-22(ok693)* dafa#3, 1 nM ISO^e^ | 74 (2.3) | 2.05 (1.04) | 89.2 (2.8) | 20.8 (9.4) | 0.015 | 7.13E-08 |
| *daf-22(ok693)* dafa#3, 0.1 nM ISO^e^ | 75.73 (4.6) | 3.78 (1.5) | 91.2 (5.5) | 37.5 (13.6) | 0.0041 | 0.0123 |
