# Supplementary material for: Larval crowding accelerates C. elegans development and reduces lifespan
Source: PLoS Genet. 2017 Apr 10;13(4):e1006717. doi: 10.1371/journal.pgen.1006717 (PMC5402976; doi:10.1371/journal.pgen.1006717)
Supplement: S15 Table — Worms were treated with dafachronic acids at 100 nM (or indicated concentrations) or mock-treated controls, using protocol B. ISO: isolation (1 worm per plate), HD; high density (50–100 worms per plate). Data shown in Fig 3E and 3F. BFBonferroni correction was used to determine significance for multiple comparisons. ePlates with EtOH (0.2% v/v). (DOCX) [file pgen.1006717.s025.docx]

| **Strain, condition** | **Time of 1^st^ egg lay [h] (STD)** | **Δ ISO-HD [h] (STD)** | **Time of 1^st^ egg of HD worms as % of ISO worms (STD)** | **Percent of wildtype  Pdda (STD)** | **P-value ISO/HD** | **P-value  N2/mutant or control/ treatment** |
| --- | --- | --- | --- | --- | --- | --- |
| N2 ISO^e^ | 69.96 (6.14) |  |  |  |  |  |
| N2 HD^e^ | 67.05 (5.5) | 2.91 (0.79) | 95.8 (7.8) | 100 (27.2) | 0.00024 |  |
| N2 dafa#3 ISO^e^ | 71.4 (5.99) |  |  |  |  |  |
| N2 dafa#3 HD^e^ | 70.85 (4.8) | 0.55 (0.87) | 99.2 (6.73) | 18.52 (29.9) | 0.69 | 0.00156^BF^ |
| *nhr-8(ok186)* ISO^e^ | 67.08 (2.44) |  |  |  |  |  |
| *nhr-8(ok186)* HD^e^ | 66.85 (2.85) | 0.23 (0.49) | 99.7 (4.24) | 8.24 (16.8) | 0.719 | 5.39E-05^BF^ |
| *nhr-8(ok186)* dafa#3 ISO^e^ | 67.65 (2.5) |  |  |  |  |  |
| *nhr-8(ok186)* dafa#3 HD^e^ | 65.8 (2.41) | 1.85 (0.38) | 97.3 (3.56) | 65.75 (13.01) | 2.82E-06 | *nhr-8* vs. *nhr-8* +dafa#3: 2.0E-05^BF^ *nhr-8* +dafa#3 vs. N2+dafa#3: 0.007^BF^ |
|  |  |  |  |  |  |  |
| N2 ISO^e^ | 68.87 (4.8) |  |  |  |  |  |
| N2 HD^e^ | 65.86 (3.5) | 3.01(0.68) | 95.6 (5.07) | 100(+-22.6) | 2.2E-07 |  |
| N2, dafa#3 ISO^e^ | 70.33 (6) |  |  |  |  |  |
| N2, dafa#3 HD^e^ | 69.62 (3.9) | 0.71(0.88) | 98.9(5.58) | 23.1(+-29.2) | 0.02 | 9.57E-05^BF^ |
| *nhr-8(ok186); daf-12(rh61 rh411)* ISO^e^ | 85.38 (8.1) |  |  |  |  |  |
| *nhr-8(ok186); daf-12(rh61 rh411)* HD^e^ | 72.5 (5.9) | 12.88(+-1.57) | 84.9 (6.9) | 345.2 (52.2) | 1.4E-10 | 1.13E-12^BF^ |
| *nhr-8(ok186); daf-12(rh61 rh411)* dafa#3, ISO^e^ | 85.69 (10.9) |  |  |  |  |  |
| *nhr-8(ok186); daf-12 (rh61 rh411*) dafa#3 HD^e^ | 73.7 (4.4) | 11.99 (2.3) | 86.01 (5.1) | 374.3 (76.4) | 7.2E-06 | *nhr-8; daf-12* +dafa#3 vs. N2+dafa#3:  1.1E-06^BF^  *nhr-8; daf-12* +dafa#3 vs. *nhr-8; daf-12*: 0.7^BF^ |
|  |  |  |  |  |  |  |
| N2 ISO^e^ | 67.75 (4.1) |  |  |  |  |  |
| N2 HD^e^ | 64.95 (3.2) | 2.8 (0.79) | 95.9 (4.7) | 100 (28.2) | 8.7E-07 |  |
| N2 ISO, dafa#3^e^ | 68.73(4.9) |  |  |  |  |  |
| N2 HD, dafa#3^e^ | 68.59(4.0) | 0.14 (0.93) | 99.8 (5.8) | 4.9 (33.2) | 0.89 | 0.00015^BF^ |
| *daf-9(dh6); daf-12(rh61rh411)* ISO^e^ | 72.64 (4.8) |  |  |  |  |  |
| *daf-9(dh6); daf-12(rh61 rh411)* HD^e^ | 69.92 (4.7) | 2.7 (0.83) | 96.3 (6.4) | 89.9 (29.6) | 0.00048 | 0.6299^BF^ |
| *daf-9(dh6); daf-12(rh61rh411)*, dafa#3 ISO^e^ | 72.88 (3.8) |  |  |  |  |  |
| *daf-9(dh6); daf-12(rh61rh411), dafa#3* HD^e^ | 70.9 (3.2) | 1.98 (0.77) | 97.28 (4.5) | 65.7 (27.5) | 0.0135 | *daf-9; daf-12*+ dafa#3 vs. N2+dafa#3: 0.0021^BF^ *daf-9; daf-12* +dafa#3 vs. *daf-9; daf-12*: 0.38^BF^ |
