# Supplementary material for: Larval crowding accelerates C. elegans development and reduces lifespan
Source: PLoS Genet. 2017 Apr 10;13(4):e1006717. doi: 10.1371/journal.pgen.1006717 (PMC5402976; doi:10.1371/journal.pgen.1006717)
Supplement: S16 Table — Mean and maximal lifespan of wild type (N2) worms grown at different population densities. "ISO" refers to assays with one worm per plate (wpp). Assays were conducted in Ithaca, NY, (1st data set, data shown in S8 Fig) and in Kiel, Germany (2nd data set). *Longest lifespan among all ISO worms in this experiment. (DOCX) [file pgen.1006717.s026.docx]

| **Condition** | **Number of plates, total number of worms** | **Mean lifespan [d] (SEM)** | **Maximal lifespan [d] (STD)** | **Median lifespan [d] (SEM)** | **Chi2, significance  (log-rank) compared to ISO** | **% of ISO mean lifespan** | **Difference to ISO mean lifespan** |
| --- | --- | --- | --- | --- | --- | --- | --- |
| N2 ISO | 74, 74 | 18.87 (0.53) | 31* | 19 (0.33) |  |  |  |
| N2 13 wpp | 5, 63 | 15.38 (0.86) | 22.6 (4.6) | 17 (0.9) | 10.045, 0.002 | 81.5 | 3.49 |
| N2 67 wpp | 4, 268 | 12.98 (0.19) | 23.5 (1) | 13 (0.2) | 81.28, 0.000 | 68.8 | 5.89 |
|  |  |  |  |  |  |  |  |
| N2 ISO | 16, 16 | 22.31(0.96) | 28* | 24 (1.34) |  |  |  |
| N2 6 wpp | 5, 30 | 21.2 (0.68) | 25.4 (1.5) | 21 (0.61) | 1.353, 0.245 | 95.01 | 1.11 |
| N2 12 wpp | 5, 60 | 20.42 (0.74) | 24.5 (0.7) | 19 (0.65) | 2.576, 0.109 | 91.52 | 1.89 |
| N2 26 wpp | 5,129 | 17.95 (0.5) | 25.4 (1.5) | 19 (0.43) | 9.853, 0.002 | 80.45 | 4.36 |
| N2 51 wpp | 5, 253 | 17.24 (0.29) | 24.4 (0.5) | 18 (0.33) | 21.498, 0.000 | 77.28 | 5.07 |
