# Supplementary material for: Larval crowding accelerates C. elegans development and reduces lifespan
Source: PLoS Genet. 2017 Apr 10;13(4):e1006717. doi: 10.1371/journal.pgen.1006717 (PMC5402976; doi:10.1371/journal.pgen.1006717)
Supplement: S17 Table — Worms were set up on experimental plates as egg (egg, lifespan protocol II) or as young adult (YA, lifespan protocol I), with or without 100 nM dafa#3 (data shown in Fig 4A–4C). "ISO" refers to assays with one worm per plate (wpp). Assays were conducted in Ithaca, NY. All plates contained ethanol (0.2% v/v). *longest lifespan among all ISO worms in this experiment. (DOCX) [file pgen.1006717.s027.docx]

| **Condition**  **Protocol I** | **Number of plates, total number of worms** | **Mean lifespan [d] (SEM)** | **Maximal lifespan [d] (STD)** | **Median lifespan [d] (SEM)** | **Chi2, significance  (log-rank) compared to ISO** | **% of ISO mean lifespan** | **Difference to ISO mean lifespan** |
| --- | --- | --- | --- | --- | --- | --- | --- |
| N2 YA ISO | 47, 47 | 11.51 (0.4) | 19* (0) | 11 (0.42) |  |  |  |
| N2 YA, 10 wpp | 6, 55 | 11.17 (0.5) | 17.83 (1.74) | 12 (0.62) | 0.07, 0.8 | 97.05 | 0.34 |
| N2 YA, 20 wpp | 6, 104 | 11.16 (0.34) | 18.3 (1.57) | 11 (0.31) | 0.01, 0.93 | 96.96 | 0.35 |
| N2 YA> 50wpp | 6, 323 | 11.27 (0.105) | 21.0 (1.27) | 11 (0.12) | 0.03, 0.87 | 97.92 | 0.24 |
| Protocol II |  |  |  |  |  |  |  |
| N2 egg, ISO | 40, 40 | 14.85 (0.67) | 25* (0) | 14 (0.79) |  |  |  |
| N2 egg, 10 wpp | 6, 57 | 13.58 (0.51) | 20.3 (1.76) | 14 (1.07) | 2.58, 0.11 | 91.45 | 1.27 |
| N2 egg, 20wpp | 6, 122 | 12.05 (0.33) | 21.0 (2.36) | 12 (0.26) | 9.85, 0.002 | 81.14 | 2.8 |
| N2 egg, >50 wpp | 6, 339 | 10.56 (0.15) | 19.7 (1.2) | 10 (0.1) | 21.5, 0.00 | 67.72 | 4.3 |
| Protocol I |  |  |  |  |  |  |  |
| N2 YA dafa#3 ISO | 33, 33 | 12.77 (0.64) | 24* (0) | 12 (0.83) |  |  |  |
| N2 YA 10 wpp dafa#3 | 6, 62 | 11.5 (0.52) | 19.3 (1.5) | 11 (0.5) | 1.38, 0.24 | 90.05 | 1.27 |
| N2 YA 20 wpp dafa#3 | 6, 124 | 11.49 (0.48) | 20.5 (2.3) | 11 (0.37) | 1.29, 0.26 | 89.98 | 1.28 |
| N2 YA >50 wpp dafa#3 | 6, 296 | 10.36 (0.2) | 19.7 (1.7) | 10 (0.25 ) | 9.65, 0.002 | 81.13 | 2.41 |
| Protocol II |  |  |  |  |  |  |  |
| N2 egg dafa#3 ISO | 24, 24 | 12.44 (0.74) | 23* (0) | 11 (0.43) |  |  |  |
| N2 egg 10 wpp dafa#3 | 6, 45 | 12.18 (0.69) | 21.5 (2.46) | 12 (0.53) | 0.04, 0.85 | 97.9 | 0.26 |
| N2 egg 20 wpp dafa#3 | 6, 124 | 11.09 (0.41) | 19.8 (2.21) | 10 (0.27) | 1.55, 0.21 | 89.1 | 1.35 |
| N2 egg >50 wpp dafa#3 | 6, 388 | 10.46 (0.19) | 21.3 (0.8) | 11 (0.22) | 3.91, 0.05 | 84.1 | 1.98 |
