# Supplementary material for: Larval crowding accelerates C. elegans development and reduces lifespan
Source: PLoS Genet. 2017 Apr 10;13(4):e1006717. doi: 10.1371/journal.pgen.1006717 (PMC5402976; doi:10.1371/journal.pgen.1006717)
Supplement: S18 Table — In all assays plates containing EtOH (0.2% v/v) were used. (DOCX) [file pgen.1006717.s028.docx]

|  | | 1 wpp,  YA | 1 wpp,  egg | 10 wpp, YA | 10 wpp, egg | 20 wpp, YA | 20 wpp, egg | 50 wpp, YA | 50 wpp, egg |
| --- | --- | --- | --- | --- | --- | --- | --- | --- | --- |
|  | 1 YA |  | 0.000 | 0.690 | 0.001 | 0.913 | 0.138 | 0.699 | 0.182 |
|  | 1wpp, egg | 0.000 |  | 0.000 | 0.144 | 0.000 | 0.000 | 0.000 | 0.000 |
|  | 10 wpp, YA | 0.690 | 0.000 |  | 0.002 | 0.734 | 0.305 | 0.999 | 0.069 |
|  | 10 wpp, egg | 0.001 | 0.144 | 0.002 |  | 0.000 | 0.017 | 0.000 | 0.000 |
|  | 20 wpp, YA | 0.913 | 0.000 | 0.734 | 0.000 |  | 0.095 | 0.656 | 0.082 |
|  | 20 wpp, egg | 0.138 | 0.000 | 0.305 | 0.017 | 0.095 |  | 0.101 | 0.000 |
|  | 50 wpp ,YA | 0.699 | 0.000 | 0.999 | 0.000 | 0.656 | 0.101 |  | 0.001 |
|  | 50 wpp, egg | 0.182 | 0.000 | 0.069 | 0.000 | 0.082 | 0.000 | 0.001 |  |
