# Supplementary material for: Larval crowding accelerates C. elegans development and reduces lifespan
Source: PLoS Genet. 2017 Apr 10;13(4):e1006717. doi: 10.1371/journal.pgen.1006717 (PMC5402976; doi:10.1371/journal.pgen.1006717)
Supplement: S19 Table — In all assays plates containing EtOH (0.2% v/v) were used. (DOCX) [file pgen.1006717.s029.docx]

|  | 1 wpp,  YA, d#3 | 1 wpp,  egg, d#3. | 10 wpp, YA, d#3 | 10 wpp,  egg, d#3 | 20 wpp, YA, d#3 | 20 wpp, egg, d#3 | 50 wpp, YA, d#3 | 50 wpp, egg, d#3 |
| --- | --- | --- | --- | --- | --- | --- | --- | --- |
| 1 wpp, YA d#3 |  | 0.731 | 0.852 | 0.380 | 0.214 | 0.562 | 0.048 | 0.014 |
| 1 wpp, egg d#3 | 0.731 |  | 0.766 | 0.237 | 0.095 | 0.256 | 0.007 | 0.002 |
| 10 wpp, YA d#3 | 0.852 | 0.766 |  | 0.331 | 0.135 | 0.375 | 0.008 | 0.002 |
| 10 wpp, egg d#3 | 0.380 | 0.237 | 0.331 |  | 0.669 | 0.853 | 0.218 | 0.067 |
| 20 wpp, YA d#3 | 0.214 | 0.095 | 0.135 | 0.669 |  | 0.409 | 0.228 | 0.066 |
| 20 wpp, egg d#3 | 0.562 | 0.256 | 0.375 | 0.853 | 0.409 |  | 0.079 | 0.010 |
| 50 wpp, YA d#3 | 0.048 | 0.007 | 0.008 | 0.218 | 0.228 | 0.079 |  | 0.402 |
| 50 wpp, egg d#3 | 0.014 | 0.002 | 0.002 | 0.067 | 0.066 | 0.010 | 0.402 |  |
