# Supplementary material for: Larval crowding accelerates C. elegans development and reduces lifespan
Source: PLoS Genet. 2017 Apr 10;13(4):e1006717. doi: 10.1371/journal.pgen.1006717 (PMC5402976; doi:10.1371/journal.pgen.1006717)
Supplement: S20 Table — In all assays plates containing EtOH (0.2% v/v) were used. (DOCX) [file pgen.1006717.s030.docx]

|  | 1 wpp,  egg, d#3 | 1 wpp,  egg | 10 wpp, egg, d#3 | 10 wpp, egg | 20 wpp, egg, d#3 | 20 wpp, egg | 50 wpp, egg, d#3 | 50 wpp, egg |
| --- | --- | --- | --- | --- | --- | --- | --- | --- |
| 1 wpp, egg, d#3 |  | 0.031 | 0.852 | 0.238 | 0.214 | 0.783 | 0.048 | 0.011 |
| 1 wpp, egg | 0.031 |  | 0.027 | 0.144 | 0.000 | 0.000 | 0.000 | 0.000 |
| 10 wpp, egg, d#3 | 0.852 | 0.027 |  | 0.257 | 0.135 | 0.573 | 0.008 | 0.002 |
| 10 wpp, egg | 0.238 | 0.144 | 0.257 |  | 0.002 | 0.017 | 0.000 | 0.000 |
| 20 wpp, egg, d#3 | 0.214 | 0.000 | 0.135 | 0.002 |  | 0.151 | 0.228 | 0.082 |
| 20 wpp, egg | 0.783 | 0.000 | 0.573 | 0.017 | 0.151 |  | 0.001 | 0.000 |
| 50 wpp, egg, d#3 | 0.048 | 0.000 | 0.008 | 0.000 | 0.228 | 0.001 |  | 0.513 |
| 50 wpp, egg | 0.011 | 0.000 | 0.002 | 0.000 | 0.082 | 0.000 | 0.513 |  |
